# Myeloma Microenvironmental TIMP1 Induces the Invasive Phenotype in Fibroblasts to Modulate Disease Progression

**DOI:** 10.3390/ijms24032216

**Published:** 2023-01-22

**Authors:** Rei Ishihara, Tsukasa Oda, Yuki Murakami, Ikuko Matsumura, Saki Watanabe, Yuta Asao, Yuta Masuda, Nanami Gotoh, Tetsuhiro Kasamatsu, Hisashi Takei, Nobuhiko Kobayashi, Nobuo Sasaki, Takayuki Saitoh, Hirokazu Murakami, Hiroshi Handa

**Affiliations:** 1 Department of Laboratory Sciences, Graduate School of Health Sciences, Gunma University, Maebashi 371-8510, Japan; 2 Laboratory of Mucosal Ecosystem Design, Institute for Molecular and Cellular Regulation, Gunma University, Maebashi 371-8510, Japan; 3 Department of Haematology, Graduate School of Medicine, Gunma University, Maebashi 371-8510, Japan; 4 Faculty of Medical Technology and Clinical Engineering, Gunma University of Health and Welfare, Maebashi 371-0823, Japan

**Keywords:** multiple myeloma, tissue inhibitor of metalloproteinase, tumour microenvironment, fibroblast

## Abstract

Tissue inhibitors of metalloproteinases (TIMPs) are endogenous matrix metalloproteinase inhibitors. TIMP1 is produced by cancer cells and has pleiotropic activities. However, its role and source in multiple myeloma (MM) are unclear. Here, we evaluated TIMP1 protein and mRNA levels in bone marrow (BM) plasma cells and assessed the effects of TIMP1 expression on fibroblast invasive capacity using three-dimensional spheroid cell invasion assays. *TIMP1* mRNA and protein levels were elevated when patients progressed from monoclonal gammopathy of undetermined significance or smouldering myeloma to MM. Furthermore, TIMP1 levels decreased at complete response and TIMP1 protein levels increased with higher international staging. *TIMP1* mRNA levels were markedly higher in extramedullary plasmacytoma and MM with t(4;14). Overall survival and post-progression survival were significantly lower in MM patients with high TIMP1 protein. Recombinant TIMP1 did not directly affect MM cells but enhanced the invasive capacity of fibroblasts; this effect was suppressed by treatment with anti-TIMP1 antibodies. Fibroblasts supported myeloma cell invasion and expansion in extracellular matrix. Overall, these results suggested that MM-derived TIMP1 induces the invasive phenotype in fibroblasts and is involved in disease progression. Further studies are required to elucidate the specific roles of TIMP1 in MM and facilitate the development of novel therapies targeting the TIMP1 pathway.

## 1. Introduction

Multiple myeloma (MM) is a malignancy of terminally differentiated monoclonal plasma cells that proliferate in the bone marrow (BM) and produce excessive monoclonal immunoglobulins. Virtually all MM progresses from an asymptomatic premalignant stage called monoclonal gammopathy of undetermined significance (MGUS); approximately 1% of patients with MGUS progress to MM each year [1,2,3]. The progression of MM involves mutations in oncogenes and tumour-suppressor genes in plasma cells [4], and such mutations may affect the tumour microenvironment such as tumour-associated macrophage (TAM) and cancer-associated fibroblast (CAF), which is involved in tumour progression and drug resistance [5,6,7].

Various tissues in the body are composed of cells and extracellular matrix (ECM). Matrix metalloproteinases (MMPs) degrade ECM components and are involved in tissue destruction in various diseases, including cancer [8]. Tissue inhibitors of metalloproteinases (TIMPs) are endogenous inhibitors of MMPs that promote fibrosis by inhibiting ECM degradation when the balance between TIMPs and MMPs favours TIMPs [9,10]. There are four subtypes of TIMPs: TIMP1–4. TIMP1 is a multifunctional protein that has pleiotropic activities independent of MMP inhibition. Moreover, TIMP1 activates intracellular signalling pathways involved in cell proliferation, differentiation, apoptosis, and cell growth inhibition [11,12,13,14]. TIMP1 is produced by several types of cancer cells and is involved in the autocrine proliferation loop [15,16,17].

The concentration of TIMP1 in the BM plasma of patients with MM is higher than that of the peripheral blood serum of healthy individuals; high TIMP1 concentrations in patients with MM are associated with poor prognosis [18,19]. TIMP1 has also been shown to have roles in osteolysis in MM-related bone lesions [20]. However, its roles and sources in MM have not been fully elucidated.

Notably, TIMP1 promotes tissue fibrosis in liver cirrhosis and lung fibrosis by influencing fibroblasts [9,21], which are essential in the cancer microenvironment, including those in haematologic malignancies [22].

Therefore, in this study, we aimed to evaluate the roles of TIMP1 expression in MM progression, especially focusing on the interaction with fibroblasts.

## 2. Results

### 2.1. TIMP1 Protein Expression Was Higher in BM Plasma from Patients with MM

**Figure 1:**
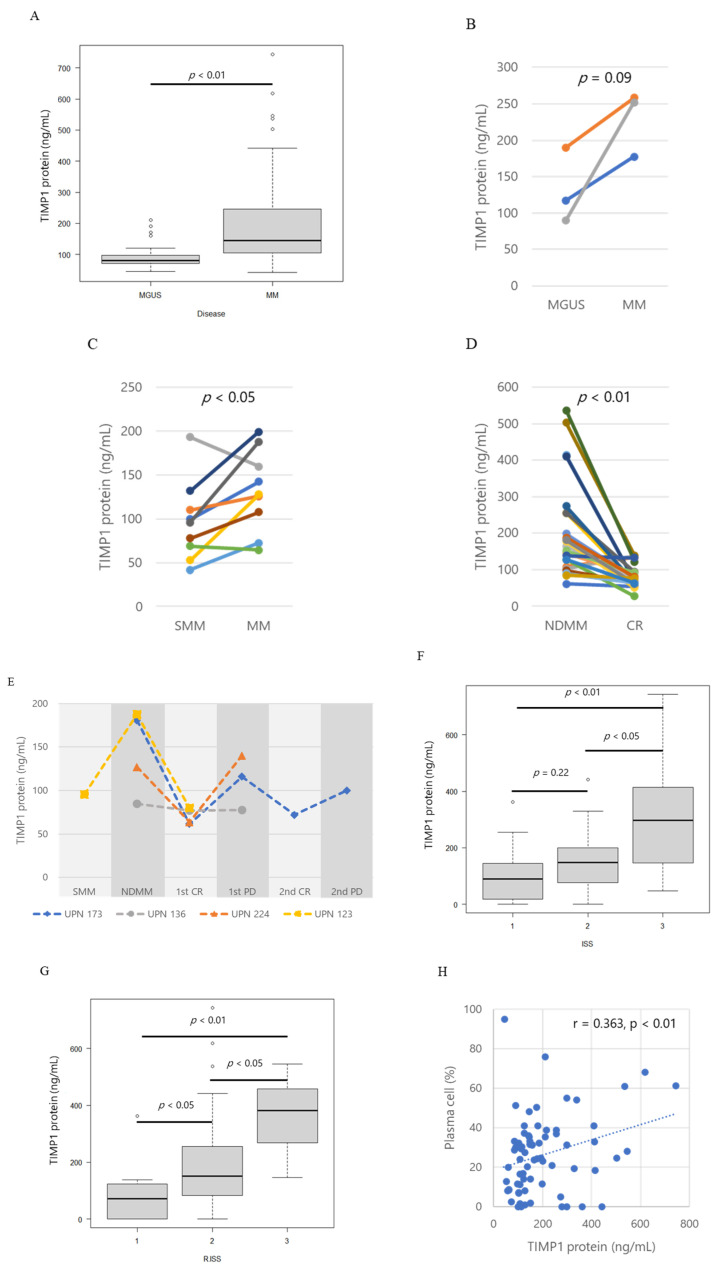
Tissue inhibitor of metalloproteinase 1 (TIMP1) protein levels. (**A**) Box plot of TIMP1 protein levels in controls, patients with monoclonal gammopathy of undetermined significance (MGUS), and patients with multiple myeloma (MM). Pairwise comparisons of TIMP1 protein levels in patients with different disease statuses. (**B**) MGUS and MM, (**C**) smouldering myeloma (SMM) and MM, and (**D**) MM at newly diagnosis (NDMM) and at complete response (CR). (**E**) TIMP1 protein levels in corresponding patients with different disease statuses; SMM, NDMM, CR, progressive disease (PD). Different colors of lines indicate corresponding patients. (**F**) Box plot of TIMP1 protein levels by international staging system (ISS) stage. (**G**) Box plot of TIMP1 protein levels by Revised ISS stage. (**H**) Correlation between TIMP1 concentrations in bone marrow plasma and plasma cell percentage in bone marrow in patients. Each dot represents an individual patient. The dotted line is the least square line.

We examined TIMP1 protein concentrations in BM plasma from patients with MGUS and MM. TIMP1 protein concentrations were significantly larger in BM plasma from patients with MM than in that from patients with MGUS (*p* < 0.001; Figure 1A). 

In the BM plasma of three patients who progressed from MGUS to MM, TIMP1 levels increased with disease progression (Figure 1B). Similarly, TIMP1 levels increased in progression from SMM to MM in seven out of nine patients (Figure 1C). TIMP1 levels decreased from the time of diagnosis to complete response (CR) in most cases (Figure 1D). In three patients (UPN173, −224, and −123), TIMP1 levels decreased in diagnosis to complete response and then increased in progressive disease (PD; Figure 1E). TIMP1 concentrations increased with higher international staging system (ISS) stages (Figure 1F) and revised ISS stages (*p* = 0.0001, *p* = 0.0013; Figure 1G). The patients at CR have no abnormal monoclonal plasma cells (i.e., CD56 + CD19-) in the bone marrows detected by flow cytometry. In the MM patients, a positive correlation was found in between the TIMP1 concentration in BM plasma and the percentage of plasma cells in BM (r = 0.363, *p* = 0.0043, Figure 1H). All these data suggest that MM tumour burden affects the TIMP1 load in the BM. 

### 2.2. TIMP1 mRNA Levels Were Higher in MM Cells Than in Control Cells

**Figure 2:**
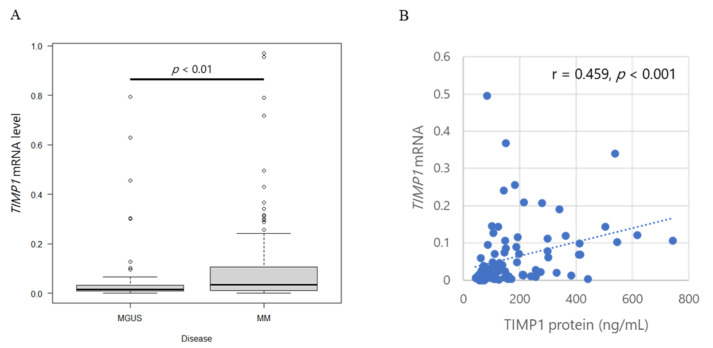

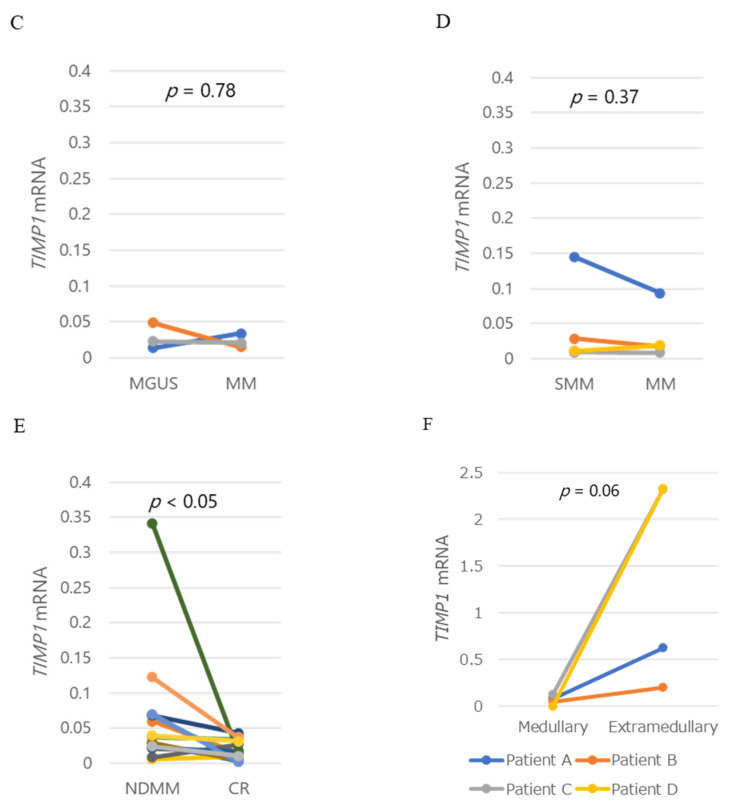
*TIMP1* mRNA levels and TIMP1 protein levels. (**A**) Box plot of *TIMP1* mRNA levels in controls and patients with MGUS or MM. (**B**) Correlation between TIMP1 concentrations in bone marrow plasma and *TIMP1* mRNA levels in bone marrow myeloma cells in patients. Each dot represents an individual patient. *TIMP1* mRNA levels in myeloma cells in bone marrow plasma from patients with (**C**) MGUS and MM, (**D**) SMM and MM, and (**E**) MM at newly diagnosis and at CR. (**F**) *TIMP1* mRNA levels in intramedullary myeloma cells and extramedullary myeloma cells. Pairwise comparisons were performed in corresponding patients. Different colors of lines indicate corresponding patients.

Our comparison between TIMP1 levels at diagnosis and at CR, positive correlation between TIMP1 and MM tumour burden, implied that myeloma cells themselves produced TIMP1. Therefore, we next examined *TIMP1* mRNA levels in purified BM plasma cells and compared *TIMP1* levels among patients with MM to patients with MGUS. *TIMP1* mRNA levels are significantly higher in MM than in MGUS (*p* < 0.01; Figure 2A), suggesting that clonal plasma cells produce higher amounts of TIMP1 during disease progression. *TIMP1* mRNA expression levels show a weak positive correlation with TIMP1 protein concentrations in BM plasma (r = 0.459, *p* < 0.001; Figure 2B).

*TIMP1* mRNA levels increased in two patients but decreased in one patient, although protein levels increased with progression from MGUS to MM in all three patients (Figure 2C). *TIMP1* mRNA levels in myeloma cells did not differ in pairwise analysis of the corresponding patients as the disease progressed from SMM to MM (Figure 2D). Notably, however, *TIMP1* mRNA levels decreased significantly from diagnosis to CR (*p* = 0.049), similar to TIMP1 protein levels in the corresponding patients (Figure 2E). Since the flow cytometric analysis at CR exhibited no abnormal clonal plasma cells in the BM, this result supports the idea that myeloma cells produce higher amounts of TIMP1. *TIMP1* mRNA levels are also markedly higher in extramedullary plasmacytoma than in BM plasma cell in corresponding patients (Figure 2F).

### 2.3. TIMP1 Expression and Cytogenetics in MM

**Figure 3:**
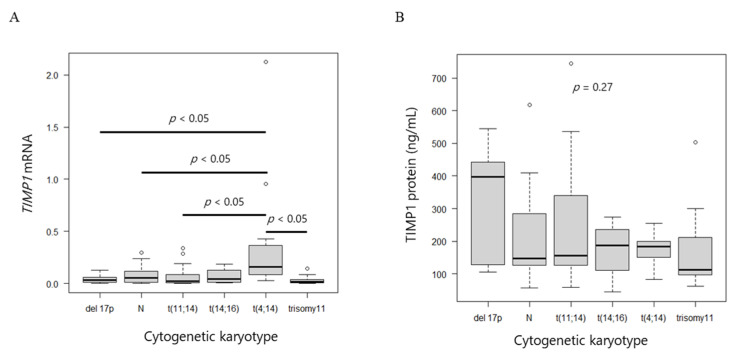
*TIMP1* mRNA and protein levels. (**A**) Box plot of *TIMP1* mRNA levels and (**B**) TIMP1 protein levels among different cytogenetic abnormalities.

*TIMP1* mRNA levels in myeloma cells and TIMP1 protein concentrations in BM plasma did not differ according to karyotype (*p* = 0.09; Figure S1A, *p* = 0.88; Figure S1B). Breakdown analysis revealed that the MM with t(4;14) expressed higher levels of *TIMP1* mRNA compared with the MM with other karyotypes (*p* = 0.0008; Figure 3A), whereas protein levels did not differ (*p* = 0.27; Figure 3B).

### 2.4. Effects of TIMP1 Expression on Patient Survival

**Figure 4:**
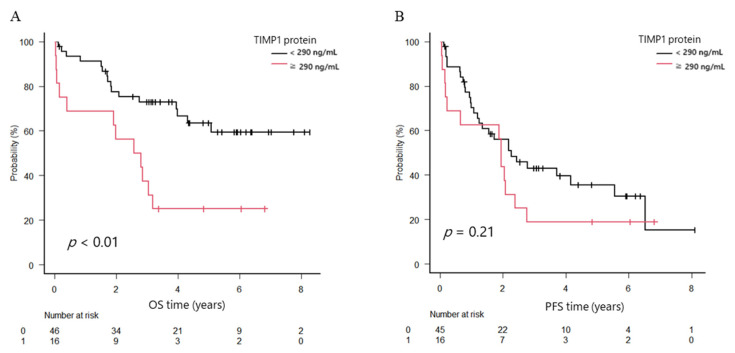

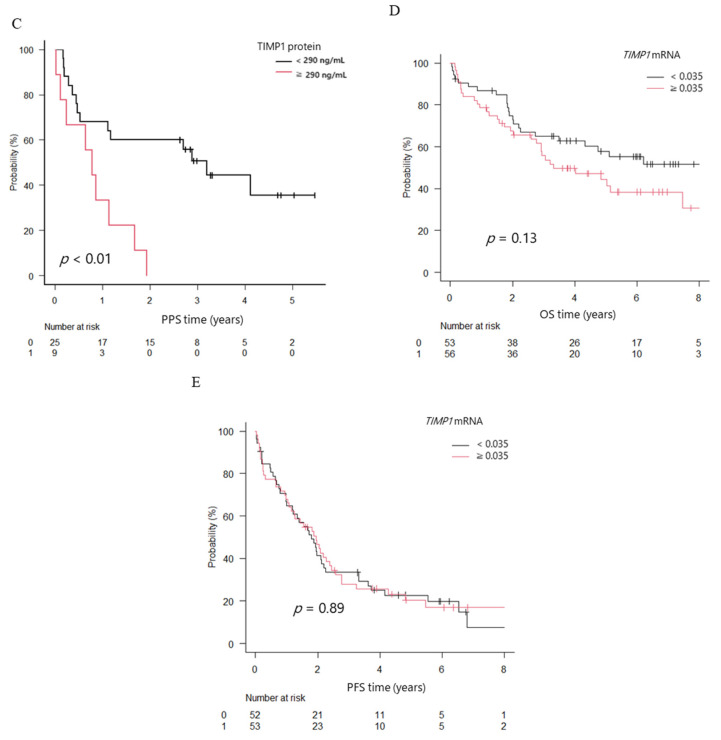
Overall survival (OS), progression-free survival (PFS), and post-progression survival (PPS) in patients with newly diagnosed multiple myeloma (NDMM) divided into two groups. (**A**) OS. (**B**) PFS. (**C**) PPS. Black line, TIMP1 protein levels in bone marrow plasma < 290 ng/mL; red line, TIMP1 protein levels ≥ 290 ng/mL. (**D**) OS. (**E**) PFS. Black line, *TIMP1* mRNA levels in bone marrow myeloma cells < 0.035; red line, *TIMP1* mRNA levels ≥ 0.035.

To clarify clinical significance of TIMP1, we analyzed patients’ survival according to TIMP1 protein and mRNA expression levels. The overall survival (OS) in the high TIMP1 protein group was 2.7 years, whereas that in the low group was not reached; the difference was significant (*p* < 0.01). On the other hand, progression-free survival (PFS) did not differ between the groups (1.9 years in the high group and 2.3 years in the low group, *p* = 0.21). Post-progression survival (PPS) was shorter (0.8 years in the high group and 2.9 years in the low group, *p* < 0.01; Figure 4A–C).

The median OS of patients with high *TIMP1* mRNA expression levels in myeloma cells (higher than median OS level of *TIMP1* in patients with MM) tended to be shorter (3.3 years versus not reached), and the 3-year OS rate was inferior (55.7% versus 65%; *p* = 0.13), although the difference was not statistically significant. The median PFS time did not differ between groups (1.79 versus 1.94 years; *p* = 0.89; Figure 4D,E).

### 2.5. TIMP1 Did Not Affect HMCL Proliferation or Drug Resistance

Since we found that high TIMP1 concentration in BM microenvironment impacted the MM patient’s prognosis, we performed an in vitro experiment using recombinant TIMP1 neutralizing anti-TIMP1 antibody and MM cell lines to clarify the role of TIMP1 on MM cells. Recombinant TIMP1 (500 ng/mL) did not affect cell proliferation or resistance to bortezomib, doxorubicin, and melphalan. Neutralizing anti-TIMP1 antibodies (4.0 µg/mL) also did not alter cell proliferation or drug resistance (Figure S2C–G). These results demonstrate that TIMP1 does not directly affect MM cell survival and proliferation.

### 2.6. TIMP1 Reinforced the Invasive Phenotype in Fibroblasts

TIMP1 has been reported to be associated with tissue fibrosis, including liver cirrhosis [23,24]. Therefore, we next studied the effects of TIMP1 expression in OUMS-36T-3F and HS-5 cells. Importantly, recombinant TIMP1 did not affect the proliferation of these two cell lines (Figure S2H).

**Figure 5:**
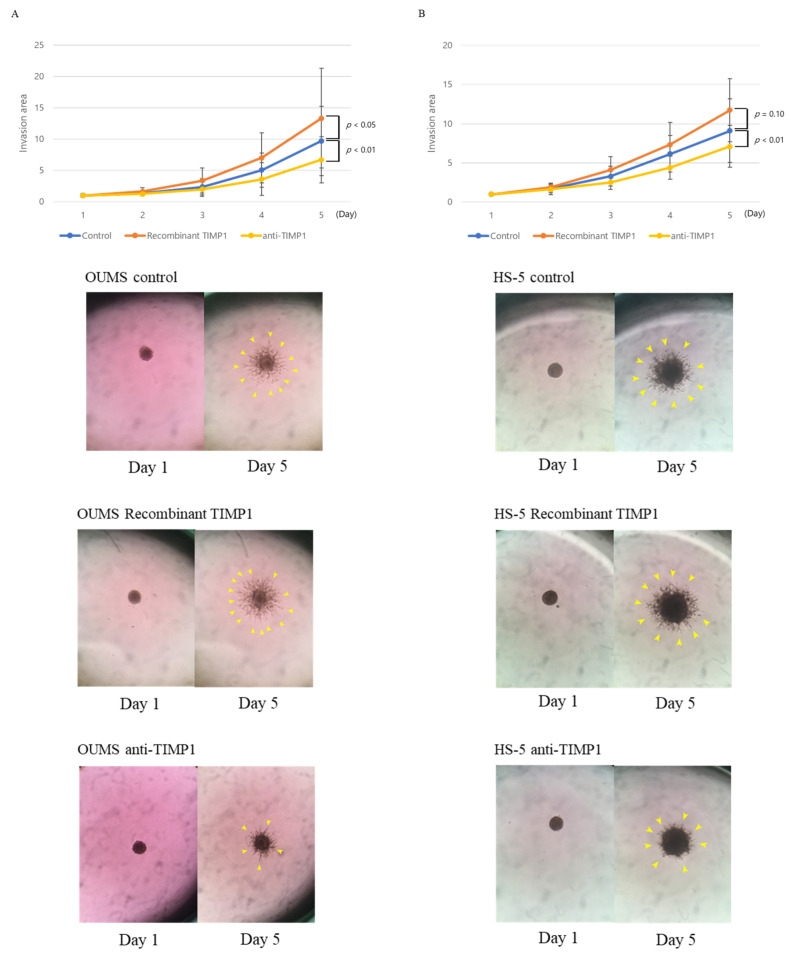
Fibroblast invasive capacity. Three-dimensional matrix invasion assays were used to evaluate the invasive capacity of (**A**) OUMS-36T-3F and (**B**) HS-5 with or without recombinant TIMP1 (0.5 µg/mL) or anti-TIMP1 antibodies (4.0 µg/mL). Blue bar, control; orange bar, recombinant TIMP1; yellow bar, anti-TIMP1 antibodies. Experiments were performed seven times. Error bars show standard deviations (SDs). Representative images of OUMS-36T-3F and HS-5 cells cultured with or without recombinant TIMP1 or anti-TIMP1 antibodies. The yellow arrows point to the spheres.

We then used 3D matrix invasion assays to examine whether TIMP1 enhanced the invasive potential of fibroblasts, thereby promoting tumour expansion or extension by supporting MM cell translocation. OUMS-36T-3F and HS-5 cells were incubated with recombinant TIMP1 for up to 96 h in a 3D matrix, and the invasion area was measured using the Fiji package over time [25]. Recombinant TIMP1 increased the invasion area of OUMS-36T-3F cells (*p* = 0.037). By contrast, treatment with an anti-TIMP1 antibody suppressed the expansion of the invasion area (*p* = 0.002; Figure 5A). Although a significant difference was not observed, HS-5 cells showed similar trends with recombinant TIMP1 (*p* = 0.10). HS-5 showed the same result with the anti-TIMP1 antibody (*p* = 0.0005; Figure 5B).

### 2.7. BM Plasma from Patients with MM Reinforced the Invasive Capacity of Fibroblasts in a TIMP1-Dependent Manner

**Figure 6:**
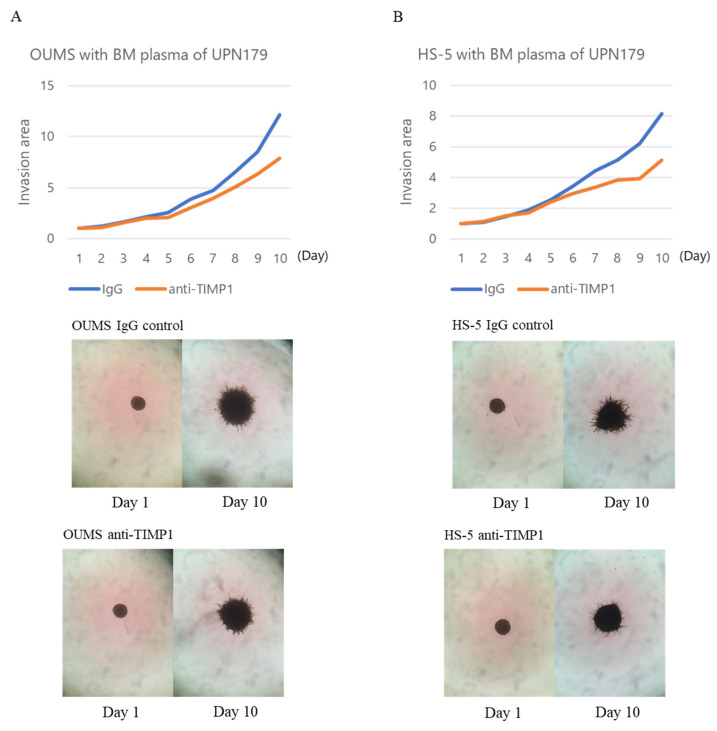
Invasive capacity of (**A**) OUMS-36T-3F and (**B**) HS-5 cells incubated with BM plasma and IgG control or neutralizing anti-TIMP1 antibodies. Blue line, IgG control; orange line, anti-TIMP1 antibodies. Representative images of OUMS-36T-3F and HS-5 cells cultured with BM plasma from patient UPN179 at diagnosis (TIMP1 protein level: 413.71 ng/mL) with IgG control or anti-TIMP1 antibodies.

Anti-TIMP1 antibody treatment decreased the invasion of OUMS-36T-3F cells induced by the patient BM plasma (UPN179, −196, and −214), compared with control nonspecific IgG treatment. Furthermore, the antibody suppressed HS-5 cell invasion induced by the BM plasma of patients UPN179, −196, −214, −264, and −317 (Figure 6A,B and Figure S3).

### 2.8. Fibroblasts Supported Myeloma Cell Invasion and Expansion in ECM

**Figure 7:**
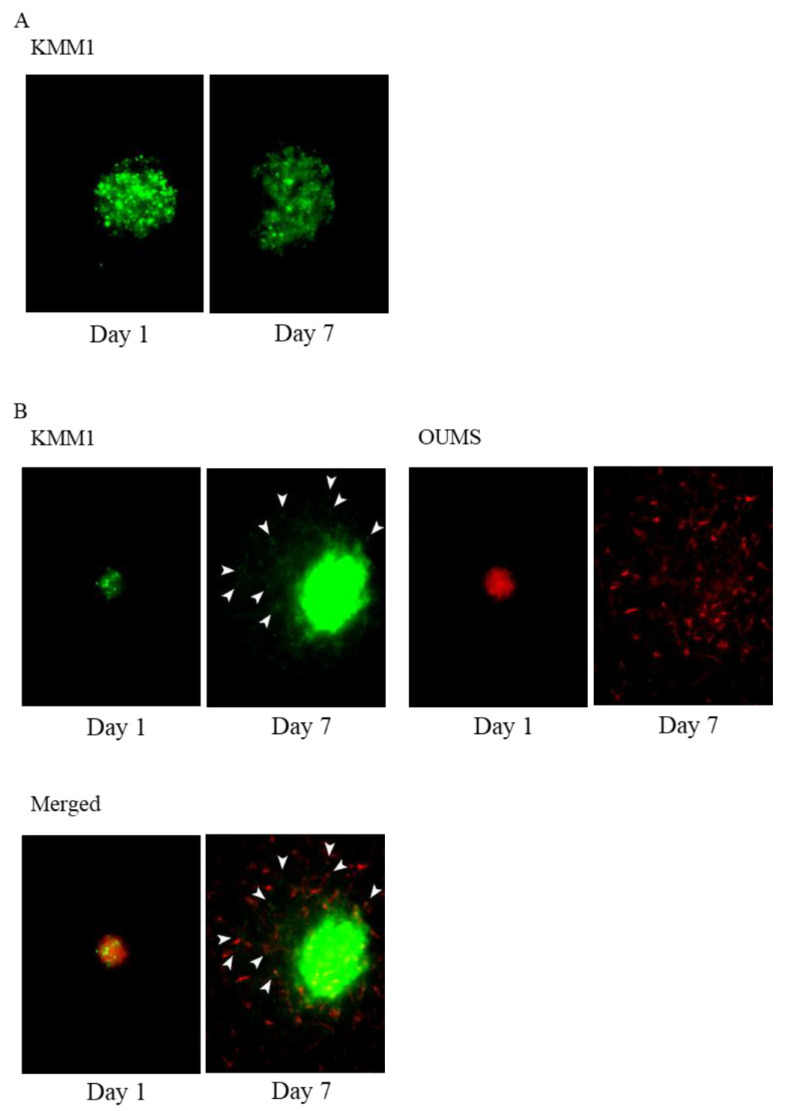
OUMS-36T-3F cells facilitate the invasion of MM cells. (**A**) Three-dimensional matrix invasion assays of KMM1 MM cells (green color stained with GFP) and (**B**) KMM1 MM cells (green color stained with GFP) with OUMS-36T-3F fibroblasts (red color stained with m-cherry). White arrows show KMM1 cells (green) migrating after OUMS-36T-3F cells (red).

We found a significant effect of TIMP1 on fibroblast invasion in the ECM as shown above; we speculated that the fibroblast could support myeloma cell invasion and expansion into ECM. To demonstrate this, we have created a co-culture system of myeloma cells and fibroblasts that emit different fluorescence. As shown in Figure 7A and Figure S4A, neither KMM1 spheroid moved or infiltrated the ECM. TMP1 did not induce MM cell invasion into the ECM (Figure S4C).

When KMM1 were cocultured with OUMS-36T-3F cells (red colour stained with m-cherry), HMCL colonies (green colour stained with green fluorescent protein [GFP]) were attracted and adhered to the branches of fibroblasts (Supplementary Video S1) and were translocated far from their original positions (Figure 7B). The same phenomenon was observed in the experiment using another myeloma cell line KMS11 and OUMS-36T-3F (Figures S4B and S5). These results suggest that microenvironmental fibroblasts or fibroblast-like cells support MM cell invasion in the matrix.

### 2.9. Transcriptome Analysis of OUMS-36T-3F Fibroblasts Treated with Recombinant TIMP1 or Anti-TIMP1 Antibodies

**Figure 8:**
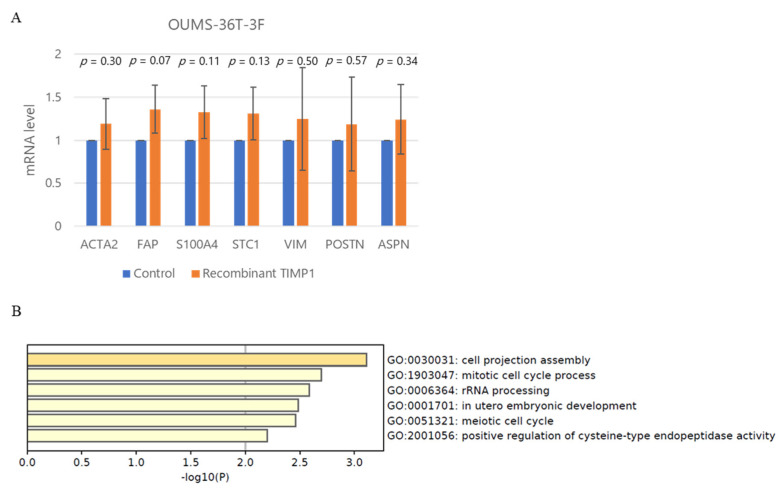

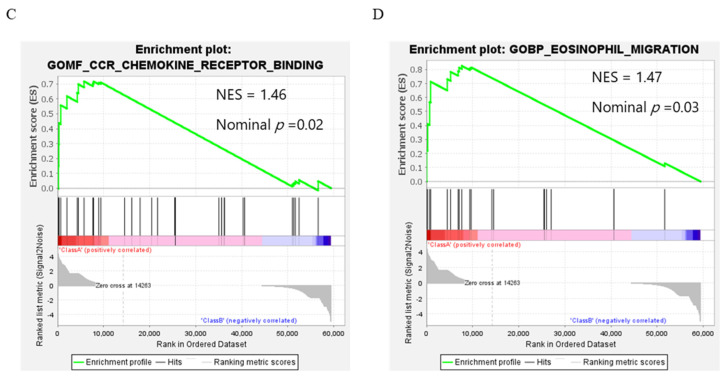
Gene expression in OUMS-36T-3F cells with or without recombinant TIMP1 (0.5 µg/mL). (**A**) q-PCR analysis of genes which are involved in cancer-associated fibroblast (CAF) formation. (**B**) Gene ontology analysis. (**C**,**D**) Gene set enrichment analysis (GSEA).

To clarify the genes involving the fibroblast invasion capacity, seven genes; *ACTA2*, *FAP*, *S100A4*, *STC1*, *VIM*, *POSTN*, *ASPN*, established to be involved in cancer-associated fibroblast (CAF) formation after treatment with TIMP1, were tested by qRT-PCR. Although the gene expressions of *ACTA2*, *FAP*, *S100A4*, and *STC1* tended to be increased by TIMP1, the increment did not reach statistical significance (Figure 8A). To find the genes involving the fibroblast transformation induced by TIMP1, whole-transcriptome analysis was performed in OUMS-36T-3F fibroblasts treated with recombinant TIMP1. In total, 88 genes were significantly upregulated and 60 genes were significantly downregulated by recombinant TIMP1 (Table S1). Gene ontology analysis revealed that the significantly upregulated genes participated in cell projection assembly in biological processes: *DNAH5*, *C2CD3*, *DYNC2I1*, *WDR11*, *SCIN*, *FGD3*, *PDGFRA*, *TIE1*, *ARID2* (GO:0030031), defined by the formation of a prolongation or process extending from a cell, for example, a flagellum or axon (Figure 8B). GSEA revealed that genes differentially expressed in response to recombinant TIMP1 were enriched in gene sets involved in chemokine receptor binding *CCL3L1*, *CCL4*, *CCL20*, *CCL5*, *CCR2*, *XCL2*, *CCL13*, *CCL27*, *CCL3*, *DEFB103A*, *CNIH4*, *CCL2*, and eosinophil migration *DAPK2*, *CCL4*, *CCL5*, *CD300A*, *CCL13*, *PTGER4*, *SCG2*, *ADAM8*, *CCL3* (Figure 8C,D and Figure S6).

## 3. Discussion

In the current study, we demonstrated that the TIMP1 protein levels in BM plasma (BM microenvironment) and the *TIMP1* mRNA expression in malignant plasma cells from patients with MM (myeloma cells) were higher than those in patients with MGUS. Furthermore, TIMP1 levels were higher in patients with advanced disease and lower in patients showing complete response. Recombinant TIMP1 promoted, whereas neutralised anti-TIMP1 antibodies inhibited fibroblast invasion, supporting the invasion of myeloma cells.

In this study, we showed that the TIMP1 protein levels in BM plasma increased with disease progression from MGUS to MM and with a higher ISS stage, suggesting that TIMP1 plays important roles in disease progression. Similarly, higher TIMP1 protein levels have been observed in BM plasma and peripheral blood serum in patients with MM compared with healthy volunteers [18,19]. However, our data comparing different stages of the disease (MGUS, SMM NDMM, and MM at complete response) and the longitudinal TIMP1 dynamics in each patient during the disease course, more clearly demonstrated that TIMP1 expression in the BM was provoked by the disease.

BM stromal cells, including mesenchymal cells, are thought to be the source of TIMP1 in the tumour microenvironment [26,27,28]. However, we found that the MM cells themselves produce TIMP1 and that the expression levels of TIMP1 increase as the disease progresses. The dynamics of TIMP1 levels during the disease course and the positive correlation between TIMP1 protein level and tumour volume in the BM, *TIMP1* mRNA levels, and TIMP1 protein levels suggest that MM cells are producers of TIMP1. These results are consistent with reports that several cancers produce TIMP1 [15,16,17]. Further studies, e.g., using single-cell RNA sequencing (scRNA-seq), are necessary to fully elucidate the primary source of *TIMP1* in MM. Indeed, Merz et al. recently reported that *TIMP1* was upregulated in myeloma cells by using scRNA-seq [29], supporting our findings. Markedly high *TIMP1* mRNA levels were observed in extramedullary plasmacytoma, which are commonly detected in the late stages of MM and are associated with poor prognosis compared with BM-MM cells in corresponding patients [30], suggesting a strong association between *TIMP1* expression and disease progression. Furthermore, the increased expression of *TIMP1* mRNA in MM with t(4;14) suggests that multiple myeloma SET domain containing protein (MMSET) may control *TIMP1*, similar to SLAM family member 7, because t(4;14) is involved in MM oncogenesis via the epigenetic regulation of MMSET expression [31]. The positive correlation between *MMSET* and *TIMP1* expression supported this hypothesis; however, further studies are required to confirm this hypothesis.

High *TIMP1* mRNA expression in breast cancer cells and high serum TIMP1 concentrations in patients with breast and gastric cancers are associated with poor prognosis [32,33,34,35,36,37]. Our result that high TIMP1 protein in the BM microenvironment was associated with worse OS is consistent with the previous studies investigating PB serum TIMP1 levels in MM [19]. Our lower cut-off value segregating prognosis suggests that TIMP1 can exert a more potent impact in limited space like in a tumour microenvironment. Since high TIMP1 clearly affected OS but not PFS, we examined survival after the first progressive disease (PD). We found that survival after PD (PPS) was clearly worse in the high TIMP1 group, which may have affected OS. Although the high *TIMP1* mRNA levels in MM cells tended to be associated with a worse prognosis, that association did not reach a statistical significance in the current study.

Then we tried to discover the role of TIMP1 in MM progression and prognosis. TIMP1 reduces drug sensitivity in breast cancer [38,39,40] and promotes the growth of Burkitt lymphoma, colon cancer, and breast cancer cells [13,15,16,17]. Additionally, high TIMP1 blood levels are associated with poor outcomes following bortezomib treatment in patients with MM [19]. However, in our study, TIMP1 did not directly affect the cell growth or drug resistance of HMCLs, and our results suggested that TIMP1 acted indirectly in MM.

The microenvironment plays important roles in the survival and proliferation of cancer cells, including MM cells, and in the development of drug resistance [3,41]. Therefore, in this study, we focused on the effects of TIMP1 on microenvironment-related cells, particularly fibroblasts, because of the strong association between TIMP1 and tissue fibrosis [9,21]. Recombinant TIMP1 treatment increased the invasion area, whereas anti-TIMP1 antibody exposure blocked the invasion of fibroblasts into the matrix, implying that TIMP1 provoked MM progression by activating and/or converting BM fibroblasts. Our results also showed that the enhancement of invasion by BM plasma from patients with NDMM was neutralised by anti-TIMP1 antibody treatment, suggesting that TIMP1 stimulated fibroblast activity in the BM of patients with symptomatic MM. Although fibroblast growth factor and transforming growth factor play pivotal roles in fibroblast growth [42,43] and are highly expressed in MM [44], TIMP1 is also critical.

Our co-culture study demonstrated that the invasion of MM cells was promoted by fibroblasts, similar to the invasive and metastatic mechanisms of other cancer cells [45]. We examined seven CAF associated genes. Although *FAP*, *A100A4*, and *STC1* repeatedly increased by TIMP1, these upregulations did not reach statistical significance. Although the individual upregulation of each gene did not reach statistical significance, we speculate that those genes acted as a group and transformed the fibroblasts. Our RNA sequencing data showed that TIMP1 enhanced the fibroblast invasive phenotype by upregulating genes related to cell projection assembly. Furthermore, GSEA also demonstrated enrichment of migration- and chemokine-related gene expression, implying that TIMP1 conferred the ability to attract myeloma cells to fibroblasts. We think that these genes functioned in collaboration. Further analysis is necessary to elucidate the roles of TIMP1 in fibroblast function.

Collectively, our findings suggest that MM-derived TIMP1 may phenotypically convert fibroblasts, similar to cancer-associated fibroblasts, and that TIMP1 may tune the MM microenvironment to promote its expansion by activating fibroblast invasion capacity. Various microenvironment-related cells contributing to MM progression, such as osteoclasts, myeloid-derived suppressor cells, and the factors that affect them, including interleukin (IL)-6 and IL-1, have been identified [46]. However, our study is the first to highlight the important roles of TIMP1 and fibroblasts in MM progression.

TIMP1 suppresses the migration of MM cells toward collagen 1 [26,47] and is involved in osteolysis in MM via the functions of osteoclasts [20]. However, the current study demonstrated a novel role of TIMP1 in MM. Further studies are needed to elucidate the regulatory mechanisms, receptors, and signal transduction pathways of TIMP1 in fibroblasts and to clarify the effects of TIMP1 on other cells in MM. MM with BM fibrosis is associated with a worse prognosis [48] and targeting CAF in BM was recently presented as a promising therapeutic strategy to prevent CAR-T resistance in MM [49]; thus, addressing these issues may contribute to the development of novel treatment approaches for refractory MM. Further studies including in vivo modelling are required to elucidate the specific roles of TIMP1 in MM and facilitate the development of novel therapies targeting the TIMP1 pathway.

## 4. Materials and Methods

### 4.1. Cell Lines

The human myeloma cell lines (HMCLs) and human fibroblast cell lines used in the current study are listed in Table 1, along with their characteristics and providers. The detailed culture conditions are provided in the Supplementary Materials.

### 4.2. Reagents

Recombinant TIMP1 (Peprotech, Rocky Hill, NJ, USA), neutralizing anti-TIMP1 antibody (AF970; R&D Systems, Minneapolis, MN, USA), anti-myeloma reagents bortezomib (Selleck Chemicals, Houston, TX, USA), doxorubicin (FUJIFILM Wako Pure Chemical Corporation, Osaka, Japan), and melphalan (LKT Laboratories, St. Paul, MN, USA) were used for all experiments.

### 4.3. Patients

In total, 159 consecutive patients with MM, 120 with newly diagnosed MM (NDMM), 25 with relapsed/refractory MM, 14 with smouldering MM (SMM), 62 with MGUS, and 31 with lymphoma without BM infiltration were enrolled in this study between January 2011 and January 2021. The demographics of MM patients are summarized in Table 2. BM aspirate samples and four biopsied extramedullary plasmacytoma samples were obtained upon diagnosis after obtaining informed consent from each patient. This study was approved by the Institutional Review Board of Gunma University Hospital under the guidelines of the Declaration of Helsinki (IRB 810 and 1295).

### 4.4. Isolation of Nucleic Acids and RNA Expression Analysis by Polymerase Chain Reaction (PCR)

Gene expression in the plasma cells of patients and in cell lines were determined by reverse transcription quantitative PCR. Primers used for the reverse transcription quantitative PCR are shown in Table 3. The detailed methods are provided in the Supplementary Materials.

### 4.5. TIMP1 Protein Concentration Assay

Plasma TIMP1 protein levels were measured using enzyme-linked immunosorbent assay kits (Quantikine; R&D Systems, Abingdon, UK) according to the manufacturer’s protocol.

### 4.6. In Vitro Cell Line Culture

HMCLs and fibroblast cell lines were treated with recombinant TIMP1 or a neutralizing anti-human TIMP1 (AF970) antibody. For drug sensitivity tests, HMCLs were cultured for 24 h in the presence of bortezomib, doxorubicin, or melphalan. Cell growth was determined by WST-8 assays (Dojindo Laboratories, Kumamoto, Japan).

### 4.7. In Vitro Cell Invasion Assays

Ninety-six-well 3D Spheroid Cell Invasion Assays (Trevigen, Gaithersburg, MD, USA) and CELLSTAR Cell-Repellent 96-well microplates (Greiner Bio-One, Kremsmünster, Austria) were used for three-dimensional (3D) invasion assays according to the manufacturer’s protocol. The detailed methods are provided in the Supplementary Materials.

### 4.8. RNA Sequencing

Whole transcriptome analysis was performed using the NextSeq 500 instrument (Illumina, San Diego, CA, USA) with a NextSeq 500/550 high-output kit v2.5 (75 cycles) (FC-404-2005; Illumina). The detailed analytical methods are provided in the Supplementary Materials. Integrated gene expression analysis was performed using gene set enrichment analysis (GSEA; Broad Institute, Cambridge, MA, USA) [50] and gene ontology analysis was performed using Metascape (version 3.5, https://metascape.org/gp/index.html#/main/step1) accessed on 27 December 2021.

### 4.9. Statistical Analysis

EZR (version 1.54; Saitama, Japan) was used for statistical analysis [51]. Results with *p* values less than 0.05 were considered statistically significant. Frequencies were evaluated using Fisher’s exact tests and continuous values were evaluated using Mann–Whitney *U* test or Kruskal–Wallis tests. Overall survival (OS) and progression-free survival (PFS) were evaluated using the Kaplan–Meier method and log-rank tests for univariate analysis. The Cox regression hazard model was used for the multivariate analysis. Cell invasion assay data were analyzed by Student’s *t* test.

## 5. Conclusions

Our findings showed that TIMP1 modulated the BM microenvironment during MM disease progression and that MM cells produced TIMP1. Moreover, TIMP1 influenced fibroblast invasion capacity, thereby contributing to MM expansion, and may be associated with a poor prognosis in patients with MM.

## Figures and Tables

**Table 1:** Human myeloma cell lines (HMCLs) and human fibroblast cell lines used in the present study.

Name	Characteristics	Providers
KMS11	HMCL, t(4;14)	Dr. Takemi Otsuki (Kawasaki Medical School, Okayama, Japan)
OPM2	HMCL, t(4;14)	Dr. Masaki Ri (Nagoya City University, Nagoya, Japan)
KMS12PE	HMCL, t(11;14)	Dr. Takemi Otsuki (Kawasaki Medical School, Okayama, Japan)
KMS26	HMCL, t(14;16)	Dr. Takemi Otsuki (Kawasaki Medical School, Okayama, Japan)
KMM1	HMCL, t(8;14)	Dr. Takemi Otsuki (Kawasaki Medical School, Okayama, Japan)
HS-5	Human fibroblast cell line	Dr. Hideto Tamura (Dokkyo Medical University, Tochigi, Japan)
OUMS-36T-3F	Human fibroblast cell line	JCRB Cell Bank (Tokyo, Japan)

**Table 2:** Patient characteristics of the 159 MM study participants.

Factor	Group	NDMM	RRMM	SMM
n		120	25	14
ASCT (%)	0	82 (73.2)	12 (48.0)	10 (100.0)
	1	30 (26.8)	13 (52.0)	0 (0.0)
Cytogenetics Karyotype (%)	del 17p	10 (9.5)	3 (13.6)	0 (0.0)
	N	28 (26.7)	4 (18.2)	4 (36.4)
	t(11;14)	27 (25.7)	7 (31.8)	1 (9.1)
	t(14;16)	5 (4.8)	0 (0.0)	0 (0.0)
	t(4;14)	14 (13.3)	4 (18.2)	3 (27.3)
	trisomy11	21 (20.0)	4 (18.2)	3 (27.3)
EMM (%)	0	57 (54.8)	16 (64.0)	10 (90.9)
	1	47 (45.2)	9 (36.0)	1 (9.1)
Gender (%)	F	57 (47.9)	12 (48.0)	8 (57.1)
	M	62 (52.1)	13 (52.0)	6 (42.9)
IgH (%)	BJ	18 (15.5)	4 (16.0)	0 (0.0)
	IgA	28 (24.1)	7 (28.0)	1 (7.1)
	IgD	3 (2.6)	0 (0.0)	0 (0.0)
	IgG	64 (55.2)	12 (48.0)	13 (92.9)
	IgM	1 (0.9)	2 (8.0)	0 (0.0)
	unknown	2 (1.7)	0 (0.0)	0 (0.0)
IgL (%)	unknown	2 (1.7)	0 (0.0)	0 (0.0)
	κ	66 (56.9)	12 (48.0)	6 (42.9)
	λ	48 (41.4)	13 (52.0)	8 (57.1)
ISS (%)	1	22 (19.3)	7 (35.0)	8 (57.1)
	2	48 (42.1)	9 (45.0)	6 (42.9)
	3	44 (38.6)	4 (20.0)	0 (0.0)
R.ISS (%)	1	13 (12.0)	3 (16.7)	5 (38.5)
	2	79 (73.1)	12 (66.7)	8 (61.5)
	3	16 (14.8)	3 (16.7)	0 (0.0)
age		70.00 [41.00, 88.00]	66.00 [42.00, 82.00]	73.50 [30.00, 81.00]
Alb		3.40 [1.90, 4.80]	3.50 [2.00, 4.90]	3.45 [2.80, 4.20]
b2MG		4.50 [1.40, 99.60]	3.50 [1.70, 123.40]	2.20 [1.30, 4.30]
LDH		173.00 [90.00, 2601.00]	211.50 [152.00, 775.00]	211.00 [122.00, 273.00]
TIMP1 Protein (ng/mL)		150.82 [44.02, 743.99]	382.17 [382.17, 382.17]	99.48 [52.72, 193.30]
*TIMP1* mRNA		0.04 [0.00, 2.12]	0.04 [0.00, 3.44]	0.02 [0.00, 0.97]

**Table 3:** Primers used in the present study.

Oligonucleotide Name	Gene	Sequence (5′→3′)
HA257212-Forward	*TIMP1*	AAGACCTACACTGTTGGCTGTGAG
HA257212-Reverse		GTCCGTCCACAAGCAATGAG
HA067803-Forward	*ACTB*	TGGCACCCAGCACAATGAA
HA067803-Reverse		CTAAGTCATAGTCCGCCTAGAAGCA
HA133460-Forward	*ACTA2*	ATTGCCGACCGAATGCAGA
HA133460-Reverse		ATGGAGCCACCGATCCAGAC
HA039683-Forward	*FAP*	CAACTGTGATGGCAAGAGCAGAA
HA039683-Reverse		TCGTGGACAGGCCGGATAA
HA037419-Forward	*S100A4*	GGGTGACAAGTTCAAGCTCAACA
HA037419-Reverse		ATCATGGCGATGCAGGACAG
HA101241-Forward	*STC1*	AGTGCTACAGCAAGCTGAATGTGTG
HA101241-Reverse		GCAGGCTTCGGACAAGTCTGTTA
HA283325-Forward	*VIM*	AACCTGGCCGAGGACATCA
HA283325-Reverse		TCAAGGTCAAGACGTGCCAGA
HA127650-Forward	*POSTN*	GCTCCTGACACAACCTGGAGA
HA127650-Reverse		AACTCCTGGTGTCAGGTGATAAAGA
HA308571-Forward	*ASPN*	CCCAAATCATTAGCAGAACTCAGAA
HA308571-Reverse		CCCTGGCTCTATCCCATTATTATCA

*TIMP1*: Tissue inhibitors of metalloproteinase 1; *ACTB*: actin beta; *ACTA2*: α-smooth muscle actin; *FAP*: Fibroblast activation protein α; *S100A4*: Fibroblast specific protein 1; *STC1*: Stanniocalcin 1; *VIM*: Vimentin; *POSTN*: Periostin; *ASPN*; Asporin.

## Data Availability

Not applicable.

## Supplementary Materials

The following supporting information can be downloaded at: https://www.mdpi.com/article/10.3390/ijms24032216/s1.
