# Supplementary material for: Myeloma Microenvironmental TIMP1 Induces the Invasive Phenotype in Fibroblasts to Modulate Disease Progression
Source: Int J Mol Sci. 2023 Jan 22;24(3):2216. doi: 10.3390/ijms24032216 (PMC9917104; doi:10.3390/ijms24032216)
Supplement: Supplementary file 1 [file ijms-24-02216-s001.zip › ijms-2133730-supplementary.pdf]

Supplementary Material

Supplemental Methods

Cell lines

The human myeloma cell lines (HMCL) used in this study and their characteristics and providers are listed in Table 1. Human bone marrow (BM) stromal fibroblast-like cell line HS-5 was obtained from Dr Hideto Tamura (Dokkyo Medical University, Tochigi, Japan). Human fibroblast cell line OUMS-36T-3F was purchased from JCRB Cell Bank (Tokyo, Japan).

The HMCLs and HS-5 cells were cultured in RPMI-1640 medium (Sigma-Aldrich, St Louis, MO, USA), OUMS-36T-3F cells were cultured in DMEM medium (FUJIFILM Wako Pure Chemical Corporation, Osaka, Japan) supplemented with 10% fetal bovine serum (Sigma-Aldrich, St Louis, MO, USA) at 37 °C in 5% CO_2.

Isolation of nucleic acids and RNA expression analysis by polymerase chain reaction (PCR)

Plasma cells were purified from BM mononuclear cells with an anti-CD138 antibody conjugated with phycoerythrin (PE) (Beckman-Coulter, Brea, CA, USA) and the Easy Sep PE positive selection kit containing anti-PE antibody conjugated with micro-magnetic beads (STEMCELL Technologies, Vancouver, BC, Canada). RNA was extracted using the mirVana RNA Isolation kit (Ambion, Austin, TX, USA) and RNeasy mini kit (Qiagen, Hilden, Germany). RNA was also extracted from paraffin-embedded tissue samples of four extramedullary plasmacytomas using a NucleoSpin total RNA FFPE XS (MACHEREY-NAGEL, Düren, Germany). Complimentary DNA (cDNA) was produced using the PrimeScript™ RT reagent kit with gDNA Eraser (TaKaRa Bio, Shiga, Japan).

Transcript levels of TIMP1 were determined by means of real-time PCR using a Power Up SYBR Green PCR Master Mix (Applied Biosystems, Foster City, CA, USA). Primers used for detection are listed in Table 3. The RNA levels were calculated using the ⊿Ct method. Expression level was normalized by actin β (ACTB) mRNA levels.

In vitro cell line culture

The HMCLs and fibroblast cell lines were treated with recombinant TIMP1 with a concentration of 0.5 ug/ml or neutralizing anti-human TIMP1 (AF970) antibody with a concentration of 4.0 ug/ml. The same concentration of non-specific human IgG was used for control. For drug sensitivity test, HMCLs were cultured for 24 h with bortezomib (0, 10, 25, 50, 100 nM), doxorubicin (0, 0.5, 1.0, 2.0, 4.0 μM), or melphalan (0, 10, 25, 50, 100 μM). Experiments were performed in triplicates. Cell growth was determined using a WST-8 assay (Dojindo Laboratory, Kumamoto, Japan) at 24, 48, and 72 h. RNA was isolated from cells incubated for 24 h with bortezomib (0, 25 nM), doxorubicin (0, 1.0 μM), or melphalan (0, 10μM), and gene expression was determined using real-time PCR. The experiments were performed in triplicates.

In vitro cell invasion assays

Cultrex 96-well 3D Spheroid Cell Invasion Assay (Trevigen, Gaithersburg, MD, USA) was used for 3D invasion assay according to the manufacturer's protocol.

Cells (2.0×10^3) suspended in 50 μL spheroid formation extracellular matrix were added to a CELLSTAR Cell-Repellent 96-well microplate (Greiner Bio-One, Kremsmünster, Austria). After

centrifugation for 3 min at 200 × *g*, cells were incubated to assemble into spheroids. After 3 days, invasion matrix (50 μL) was added onto each well, and plates were incubated for 1 h at 37 °C to enhance gel formation. Culture medium with or without reagents (100μL) were added, and then the plates were further incubated in a 5% CO_2 incubator at 37 °C for 96 hours. Invasion of cells into surrounding matrix was observed and photographed every 24 h. The spheroid area was measured using a Fiji package (ImageJ 1.53q; Wayne Rasband and contributors, National Institutes of Health, USA)[25].

RNA sequencing

Whole transcriptome analysis of isolated RNAs was performed using the NextSeq 500 (Illumina, San Diego, CA, USA) with NextSeq 500/550 high-output kit v2.5 (75 cycles) (FC-404-2005; Illumina). RNA integrity numbers were confirmed to be higher than seven using the Agilent RNA6000 Pico kit (5067-1513) in the Agilent 2100 Bioanalyzer (Agilent, Santa Clara, CA, USA). To construct the RNA library, we used the NEBNext Ultra Directional RNA Library Prep Kit for Illumina (E7420; NEB, Ipswich, MA, USA), NEBNext Multiplex Oligos for Illumina (E7335 or 7500), and Agencourt AMPure XP (A63881; Beckman Coulter, Brea, CA, USA) following the respective manufacturers' protocols. The quality of the amplified cDNA library was determined using the Agilent 2100 Bioanalyzer and the high-sensitivity DNA kit (5067-4626; Agilent). RNA-Seq was performed, and raw data were aligned against the human genome reference (hg38) using a DRAGEN RNA pipeline application (provided by Illumina), and differential expression was analyzed using the DRAGEN Differential Expression application (Illumina). Gene set enrichment

analysis (GSEA) (Broad Institute, Cambridge, MA, USA) was performed to quantify gene expression levels and determine the enrichment of specific gene sets in the RNA-Seq data [50].

Supplemental Results

TIMP1 did not directly affect myeloma cells to induce either cell proliferation or drug resistance

Five myeloma cell lines, KMS11 with t(4;14), which expresses a lower amount of TIMP1, OPM2 with t(4;14) and KMS12PE with t(11;14), which expresses a relatively higher amount of TIMP1, KMS26 with t(14;16), which expresses a relatively lower amount of TIMP1, KMM1 derived from extramedullary disease, which expresses a lower amount of TIMP1, were tested (Figure S2A), and their TIMP1 protein concentrations were found to be low (Figure S2B). They were treated with recombinant TIMP1 (0.5 μg/mL), however, the cell proliferation or survival did not change (Figure S2C). To block the autocrine effects of TIMP1 produced by myeloma cell lines, we added neutralizing anti-TIMP1 antibody (4.0 μg/mL) or IgG control; however, the proliferation or survival did not change (Figure S2D). Since TIMP1 was shown not to be involved in cell growth, implication to drug resistance was examined. Drug resistance was determined in the five myeloma cell lines with or without recombinant TIMP1 (0.5 μg/mL) treated with bortezomib, doxorubicin, and melphalan. As shown in Figure S2E, F, and G, addition of TIMP1 did not affect the cell survival of cells treated with these chemotherapeutic drugs.

Effects of TIMP1 on fibroblast cell line proliferation

Next, we studied whether TIMP1 affects microenvironmental cells, especially fibroblasts, since the role of cancer-associated fibroblasts (CAF) has received considerable attention and TIMP1 has been reported to be associated with tissue fibrosis such as liver cirrhosis[25,26]. To examine the

effect of TIMP1 on fibroblast, we used two fibroblast models: immortalized human fibroblast cell line OUMS-36T-3F and human bone marrow stromal fibroblast-like cell line HS-5. Treatment with either recombinant TIMP1 did not affect cell proliferation/survival of these two cell lines. (Figure S2H).

Figure S1

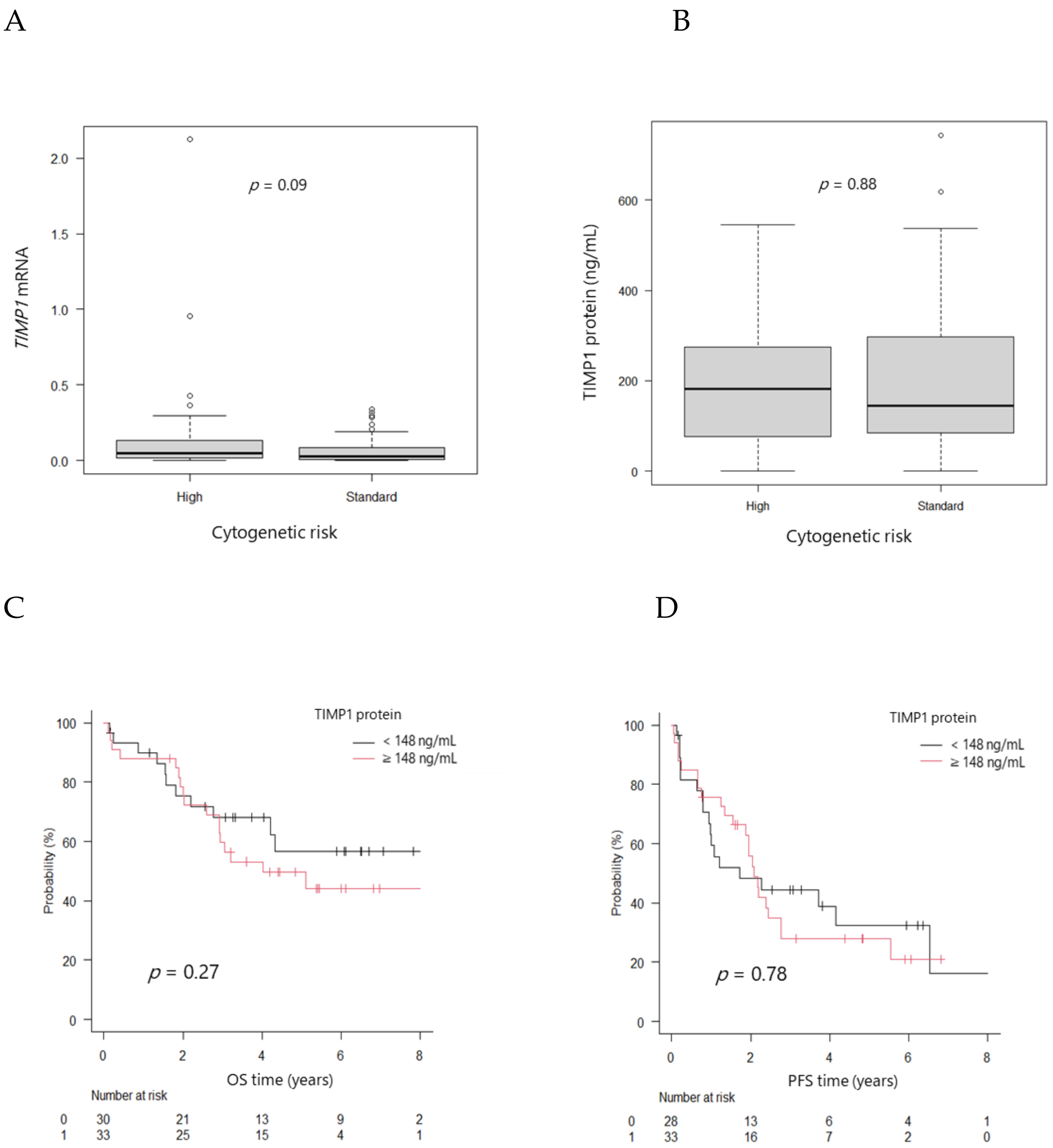

Figure S1. TIMP1 mRNA levels determined using RQ-PCR and TIMP1 protein levels determined by ELISA in patients with MM. (A) Box plot of TIMP1 mRNA levels and (B) TIMP1 protein levels in patients with MM with or without a high-risk cytogenetic karyotype. (C) Overall survival (OS) and (D) progression-free survival (PFS) in patients with newly diagnosed multiple myeloma (NDMM) divided into two groups. Black line, TIMP1 protein levels in bone marrow plasma < 148 ng/mL; red line, TIMP1 protein levels ≥ 148 ng/mL.

Figure S2

A

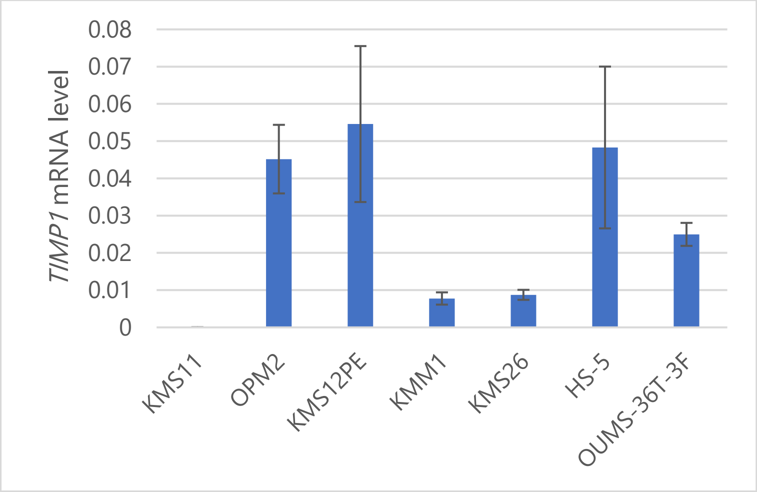

TIMP1 mRNA level
0.08
0.07
0.06
0.05
0.04
0.03
0.02
0.01
0
KMS11
OPM2
KMS12PE
KMM1
KMS26
HS-5
OUMS-36T-3F

B

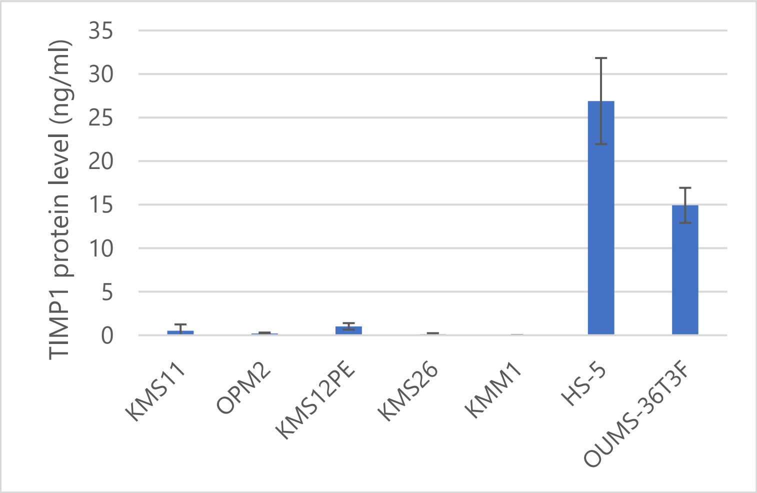

TIMP1 protein level (ng/ml)
35
30
25
20
15
10
5
0
KMS11
OPM2
KMS12PE
KMS26
KMM1
HS-5
OUMS-36T3F

C

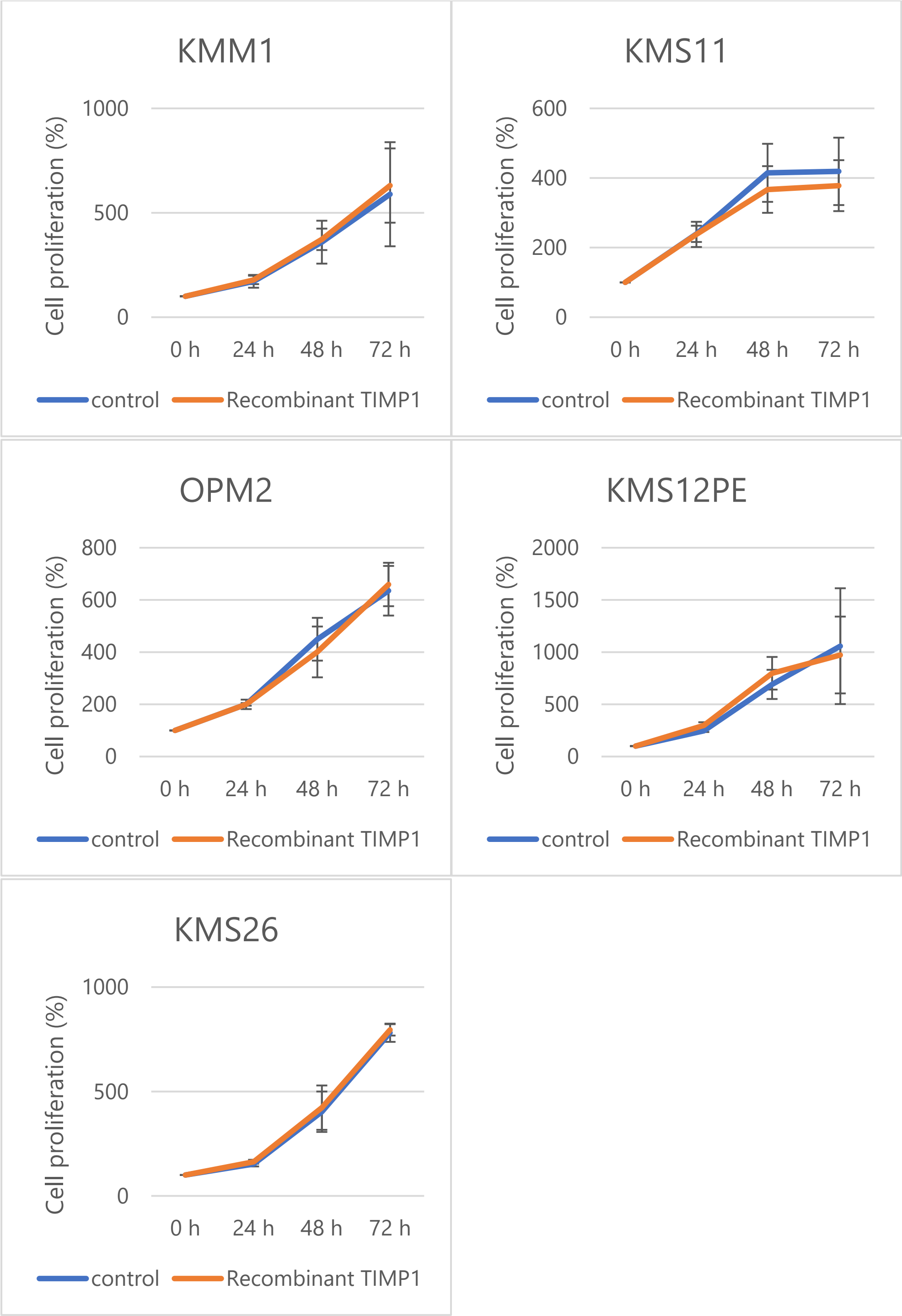
KMM1
Cell proliferation (%)
1000
500
0
0 h
24 h
48 h
72 h
control
Recombinant TIMP1
KMS11
Cell proliferation (%)
600
400
200
0
0 h
24 h
48 h
72 h
control
Recombinant TIMP1
OPM2
Cell proliferation (%)
800
600
400
200
0
0 h
24 h
48 h
72 h
control
Recombinant TIMP1
KMS12PE
Cell proliferation (%)
2000
1500
1000
500
0
0 h
24 h
48 h
72 h
control
Recombinant TIMP1
KMS26
Cell proliferation (%)
1000
500
0
0 h
24 h
48 h
72 h
control
Recombinant TIMP1

D

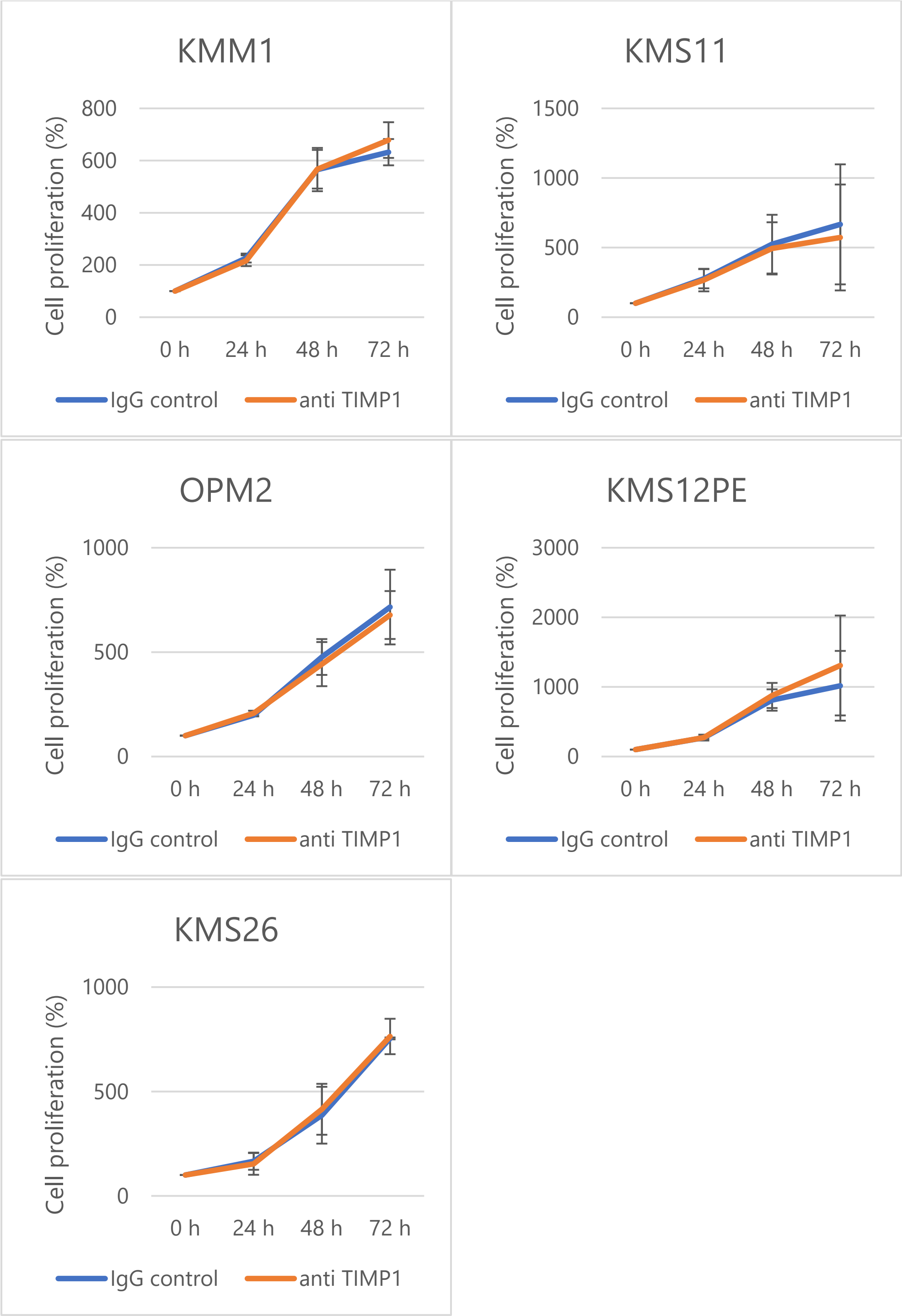

KMM1
Cell proliferation (%)
800
600
400
200
0
0 h
24 h
48 h
72 h
IgG control
anti TIMP1
KMS11
Cell proliferation (%)
1500
1000
500
0
0 h
24 h
48 h
72 h
IgG control
anti TIMP1
OPM2
Cell proliferation (%)
1000
500
0
0 h
24 h
48 h
72 h
IgG control
anti TIMP1
KMS12PE
Cell proliferation (%)
3000
2000
1000
0
0 h
24 h
48 h
72 h
IgG control
anti TIMP1
KMS26
Cell proliferation (%)
1000
500
0
0 h
24 h
48 h
72 h
IgG control
anti TIMP1

E

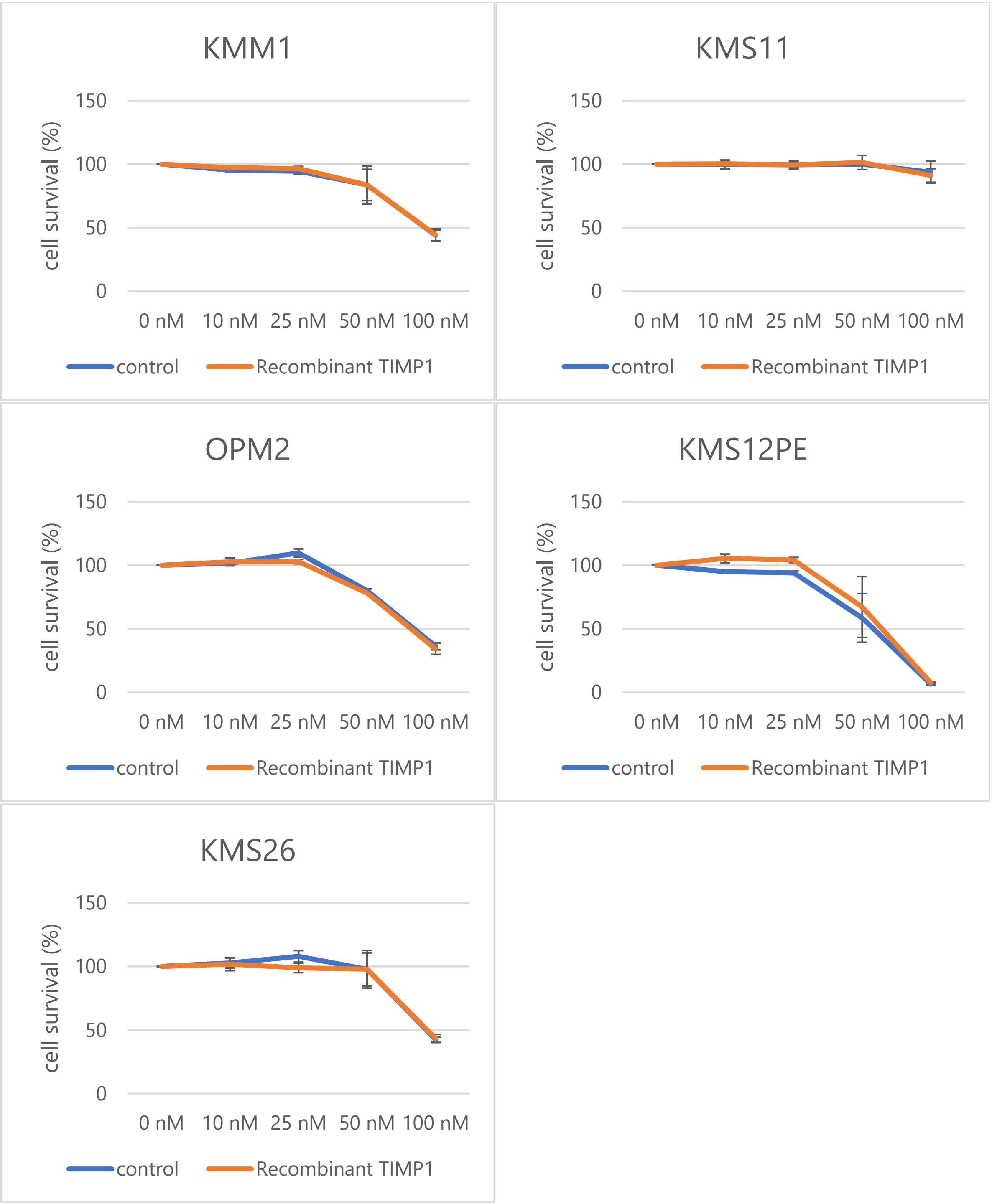
KMM1
KMS11
OPM2
KMS12PE
KMS26
cell survival (%)
150
100
50
0
0 nM
10 nM
25 nM
50 nM
100 nM
control
Recombinant TIMP1

F

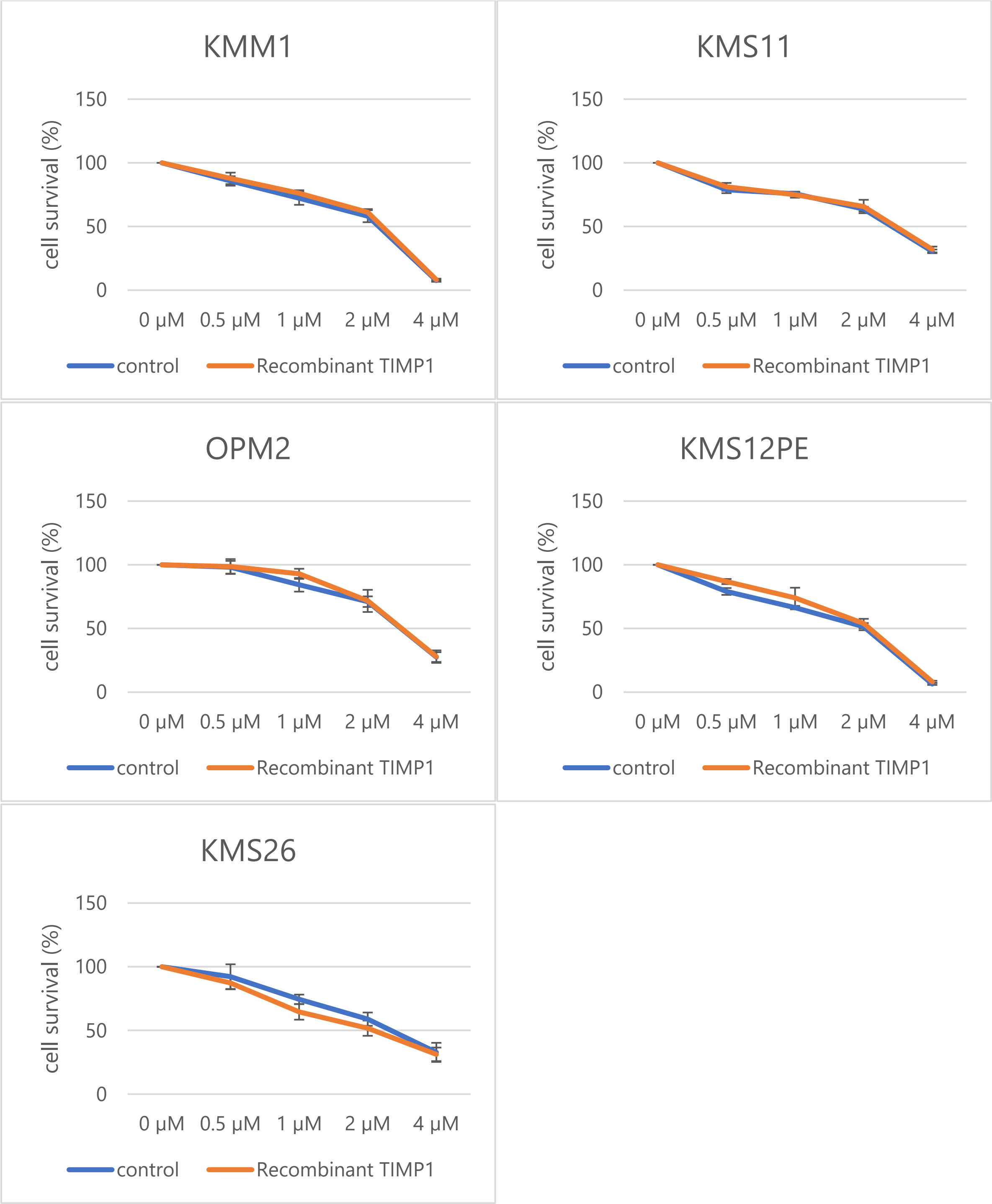
KMM1
KMS11
OPM2
KMS12PE
KMS26
cell survival (%)
150
100
50
0
0 μM
0.5 μM
1 μM
2 μM
4 μM
control
Recombinant TIMP1

G

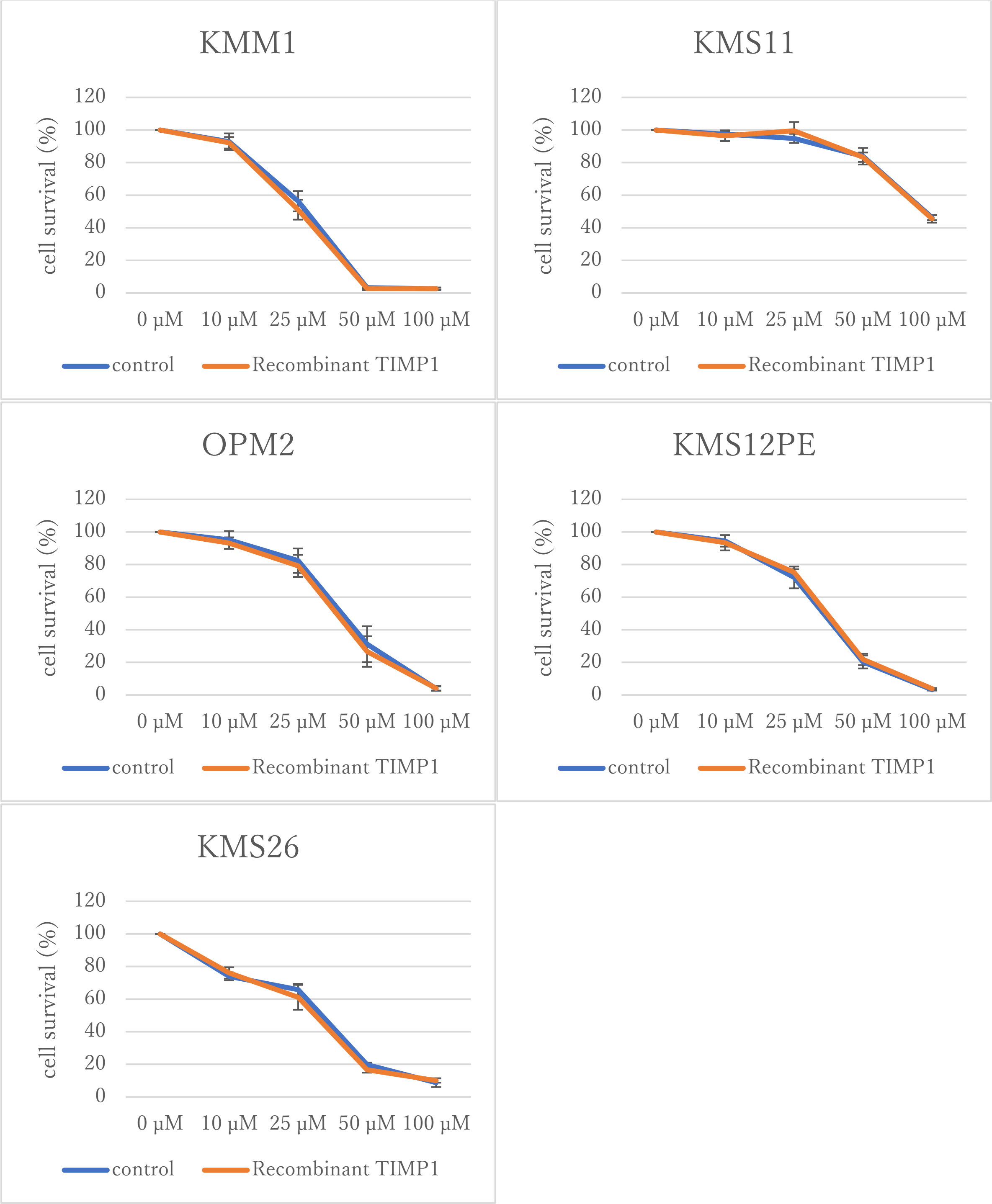
KMM1
KMS11
OPM2
KMS12PE
KMS26
cell survival (%)
120
100
80
60
40
20
0
0 μM
10 μM
25 μM
50 μM
100 μM
control
Recombinant TIMP1

H

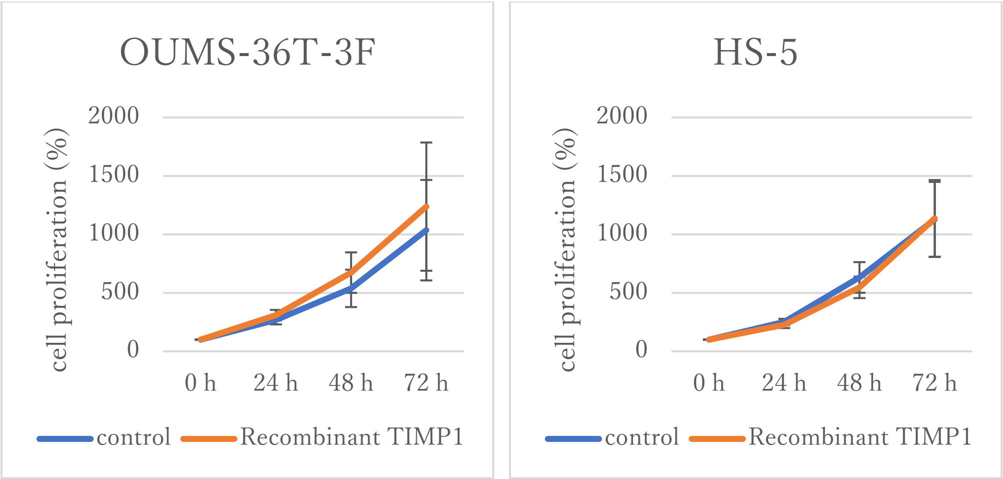

Figure S2 TIMP1 mRNA levels determined using RQ-PCR in myeloma cell lines, human immortalized fibroblast cell line OUMS-36T-3F, and human bone marrow stromal cell line HS-5. TIMP1 protein levels in cell line culture medium was determined by ELISA, and cell growth and drug resistance was determined by WST-8 assay. (A) TIMP1 mRNA levels. Error bars show the standard deviation (SD) in triplicated experiments. (B) TIMP1 protein levels. Error bars show the SD in triplicated experiments. Effects of TIMP1 in five multiple myeloma (MM) cell lines. (C) Cell growth curve with or without recombinant TIMP1 (0.5 μg/ml): blue line, control; orange line, with recombinant TIMP1. (D) Cell growth curve with IgG control or anti-TIMP1 antibodies (4.0 μg/ml): blue line, with IgG control; orange line, with anti-TIMP1 antibodies. Error bars show the SD in triplicated experiments. Drug resistance in five MM cell lines with or without recombinant TIMP1 (0.5 μg/ml) treated with bortezomib (0, 10, 25, 50, 100 nM), doxorubicin (0, 0.5, 1, 2, 4 μM), and melphalan (0, 10, 25, 50 100 μM). (E) Cell growth with or without recombinant TIMP1 under treatment with bortezomib, (F) doxorubicin, and (G) melphalan: blue line, control; orange line, with recombinant TIMP1. Error bars show the SD in triplicated experiments. Effects of TIMP1 in human immortalized fibroblast cell line OUMS-36T-3F and human bone marrow stromal cell line

HS-5. (H) Cell growth curve with or without recombinant TIMP1 (0.5 μg/ml): blue line, control; orange line, with recombinant TIMP1. Error bars show the SD in triplicated experiments.

Figure S3

A

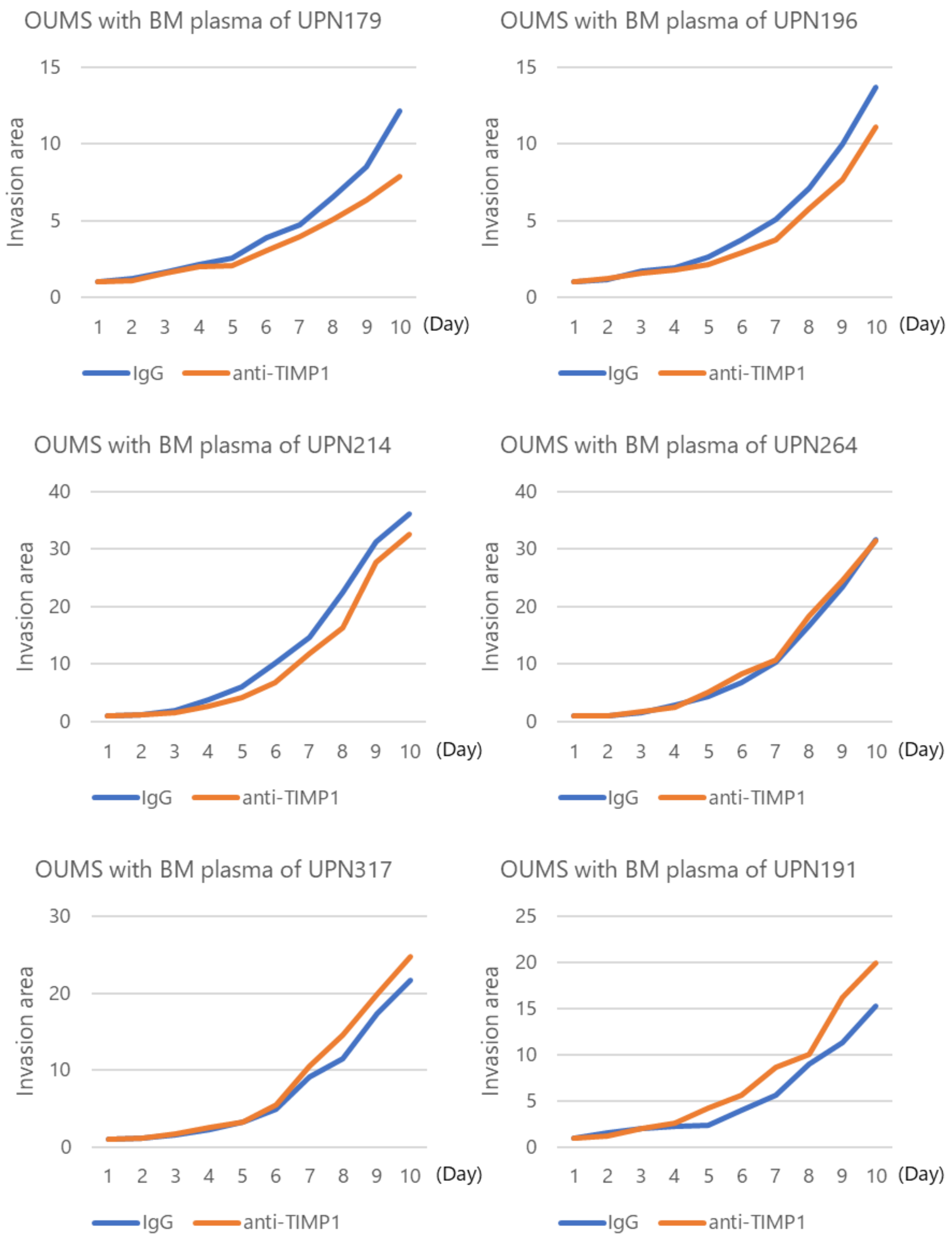
OUMS with BM plasma of UPN179
OUMS with BM plasma of UPN196
OUMS with BM plasma of UPN214
OUMS with BM plasma of UPN264
OUMS with BM plasma of UPN317
OUMS with BM plasma of UPN191
Invasion area
(Day)
IgG
anti-TIMP1

B

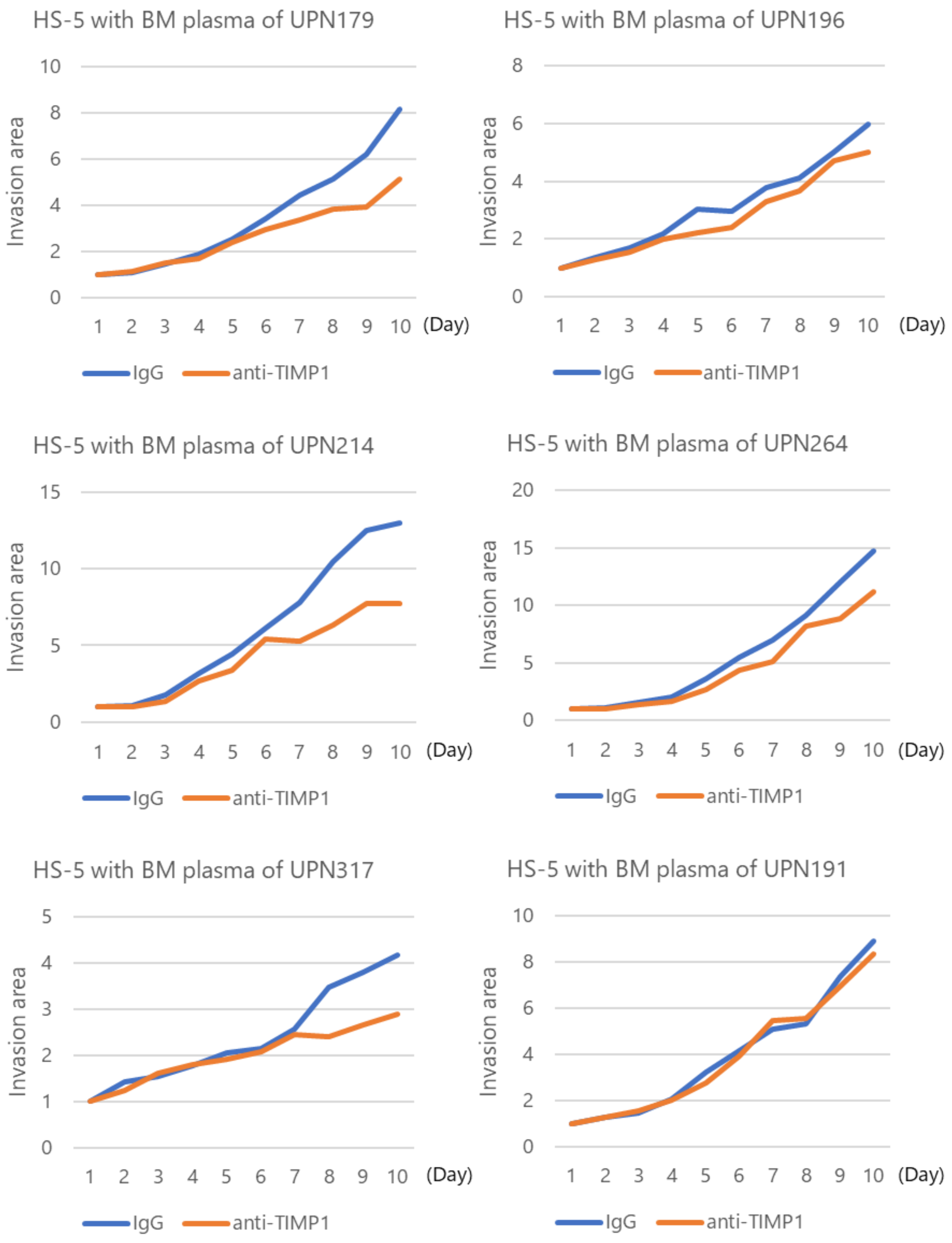
HS-5 with BM plasma of UPN179
HS-5 with BM plasma of UPN196
HS-5 with BM plasma of UPN214
HS-5 with BM plasma of UPN264
HS-5 with BM plasma of UPN317
HS-5 with BM plasma of UPN191
Invasion area
(Day)
IgG
anti-TIMP1

C

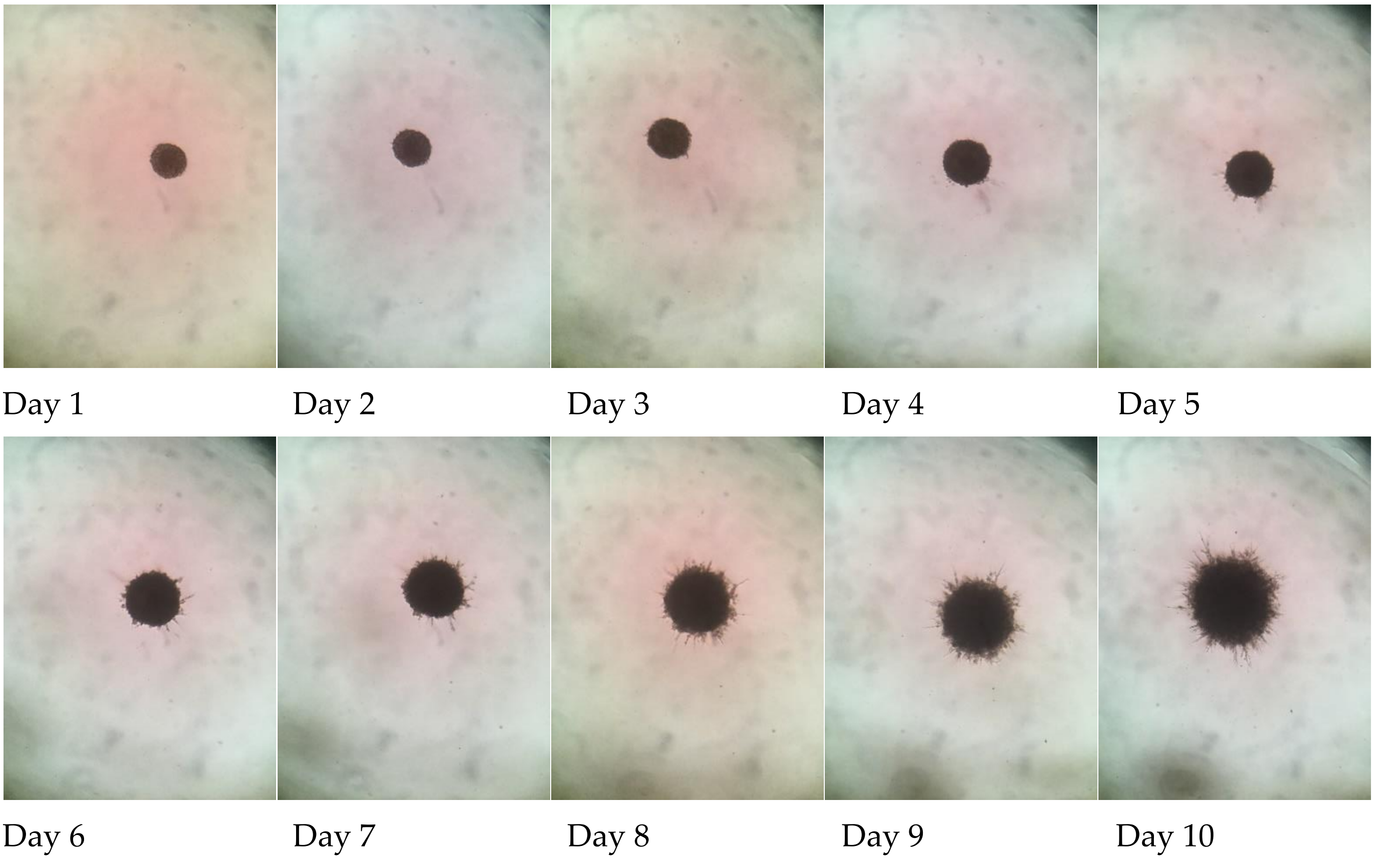
Day 1
Day 2
Day 3
Day 4
Day 5
Day 6
Day 7
Day 8
Day 9
Day 10

D

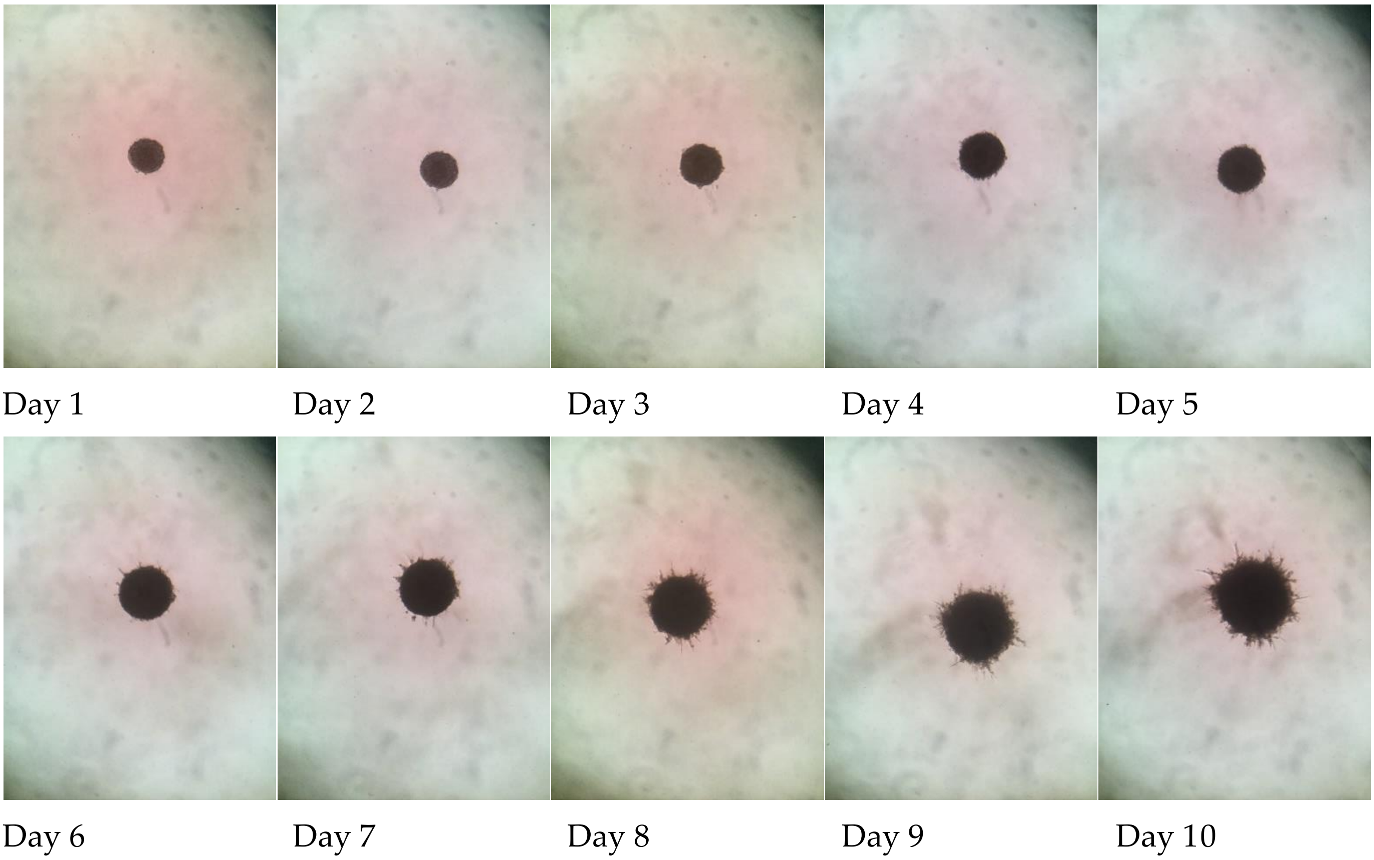
Day 1
Day 2
Day 3
Day 4
Day 5
Day 6
Day 7
Day 8
Day 9
Day 10

E

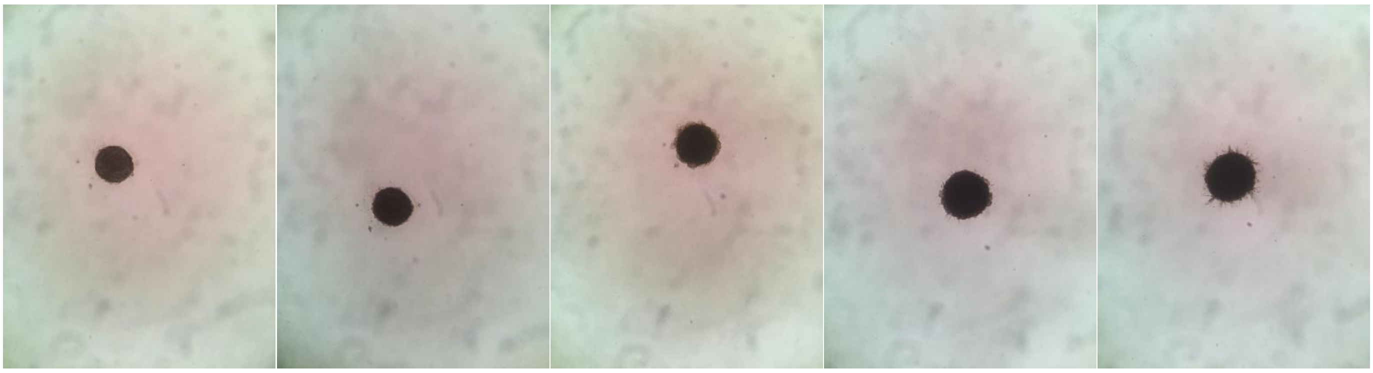

Day 1 Day 2 Day 3 Day 4 Day 5

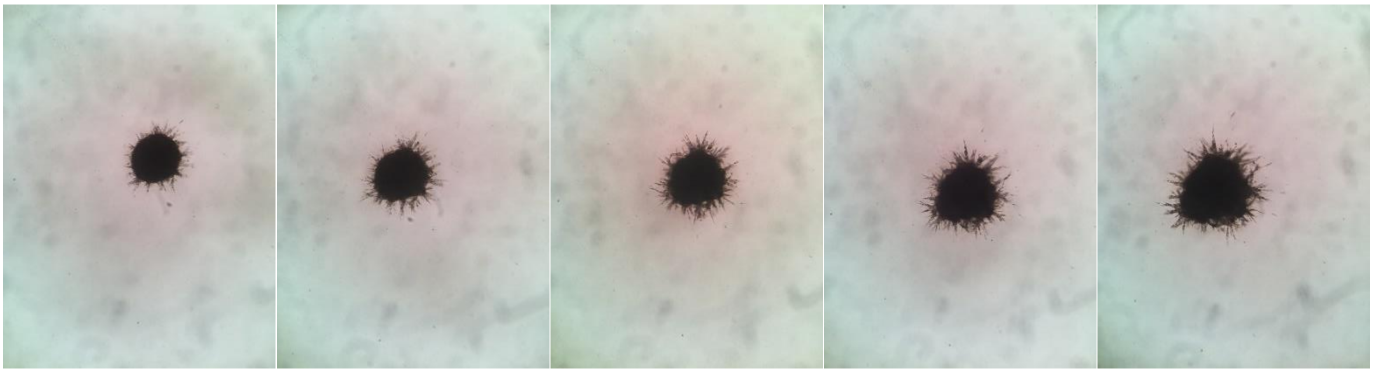

Day 6 Day 7 Day 8 Day 9 Day 10

F

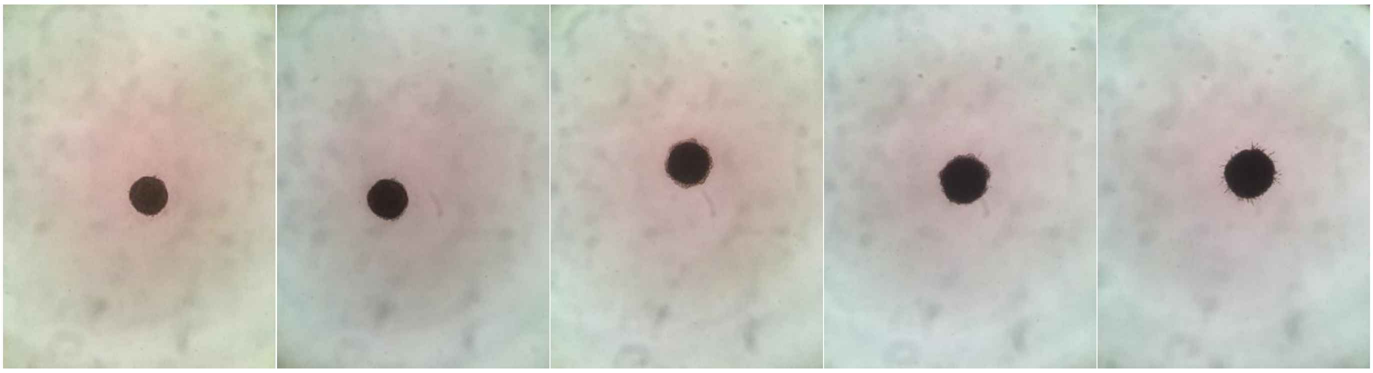

Day 1 Day 2 Day 3 Day 4 Day 5

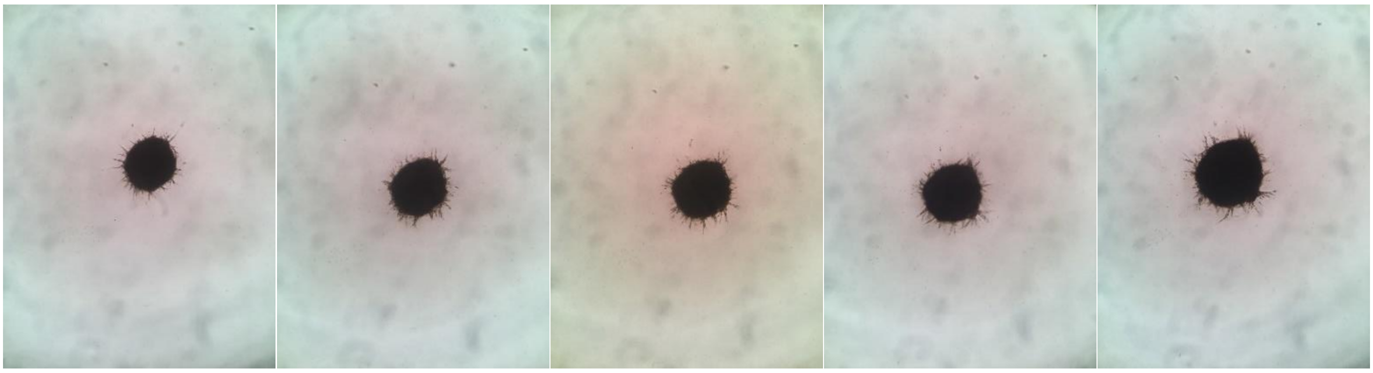

Day 6 Day 7 Day 8 Day 9 Day 10

Figure S3 Invasive capacity of the cell lines determined by 3D matrix invasion assays. Invasive capacity of (A) OUMS-36T-3F and (B) HS-5 cells incubated with BM plasma and IgG control or neutralizing anti-TIMP1 antibodies. Blue line, IgG control; orange line, anti-TIMP1 antibodies. Representative images of OUMS (C) with UPN179 (TIMP1 protein level 413.71 ng/mL) with IgG control, (D) with UPN179 with neutralizing anti-TIMP1; Images of HS-5 (E) with UPN179 with IgG control, (F) with UPN179 with neutralizing anti-TIMP1.

Figure S4

A

KMM1

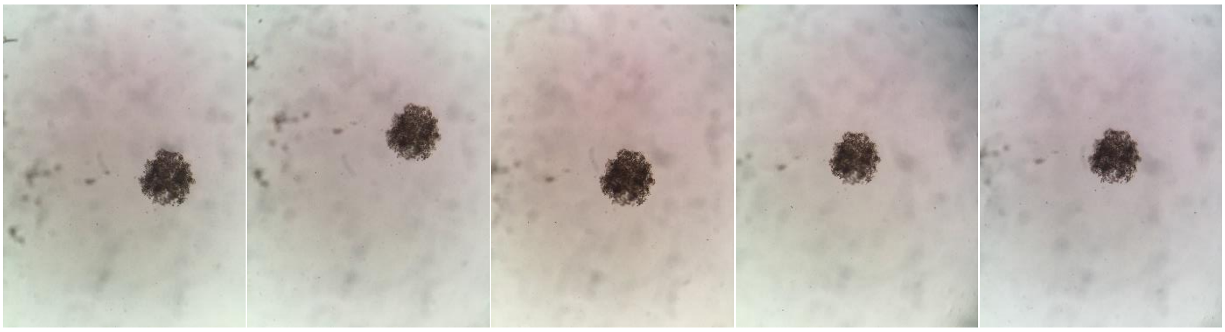

Day 1 Day 2 Day 3 Day 4 Day 5

B

KMS11

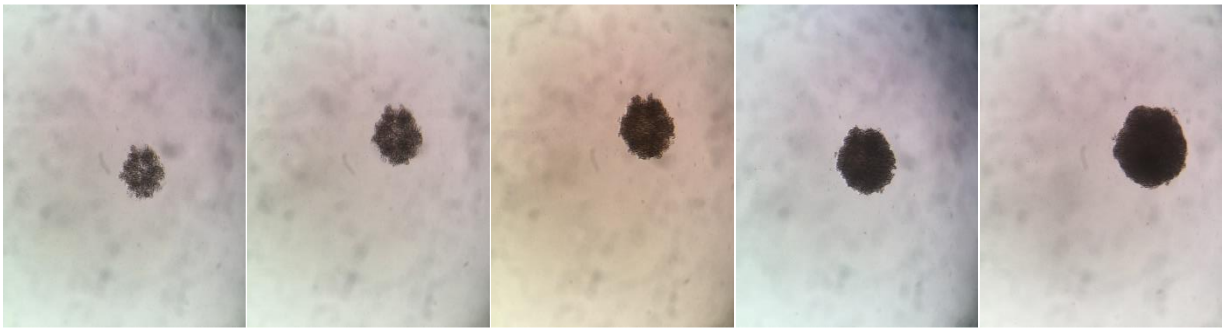

Day 1 Day 2 Day 3 Day 4 Day 5

C

KMM1 KMM1 with Recombinant TIMP1

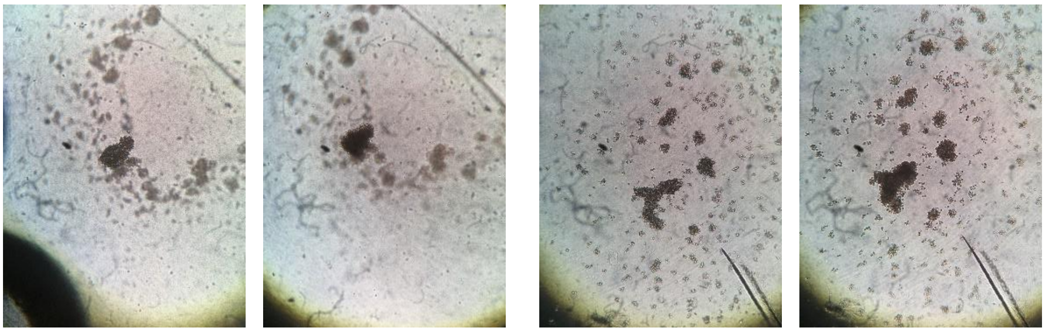

Day 1 Day 5 Day 1 Day 5

Figure S4 Invasive capacity of KMS11 and KMM1 determined by 3D matrix invasion assays.

Images of (A) KMM1, (B) KMS11, (C) KMM1 with or without Recombinant TIMP1 (0.5 μg/ml) from day 1 to day 5.

Figure S5

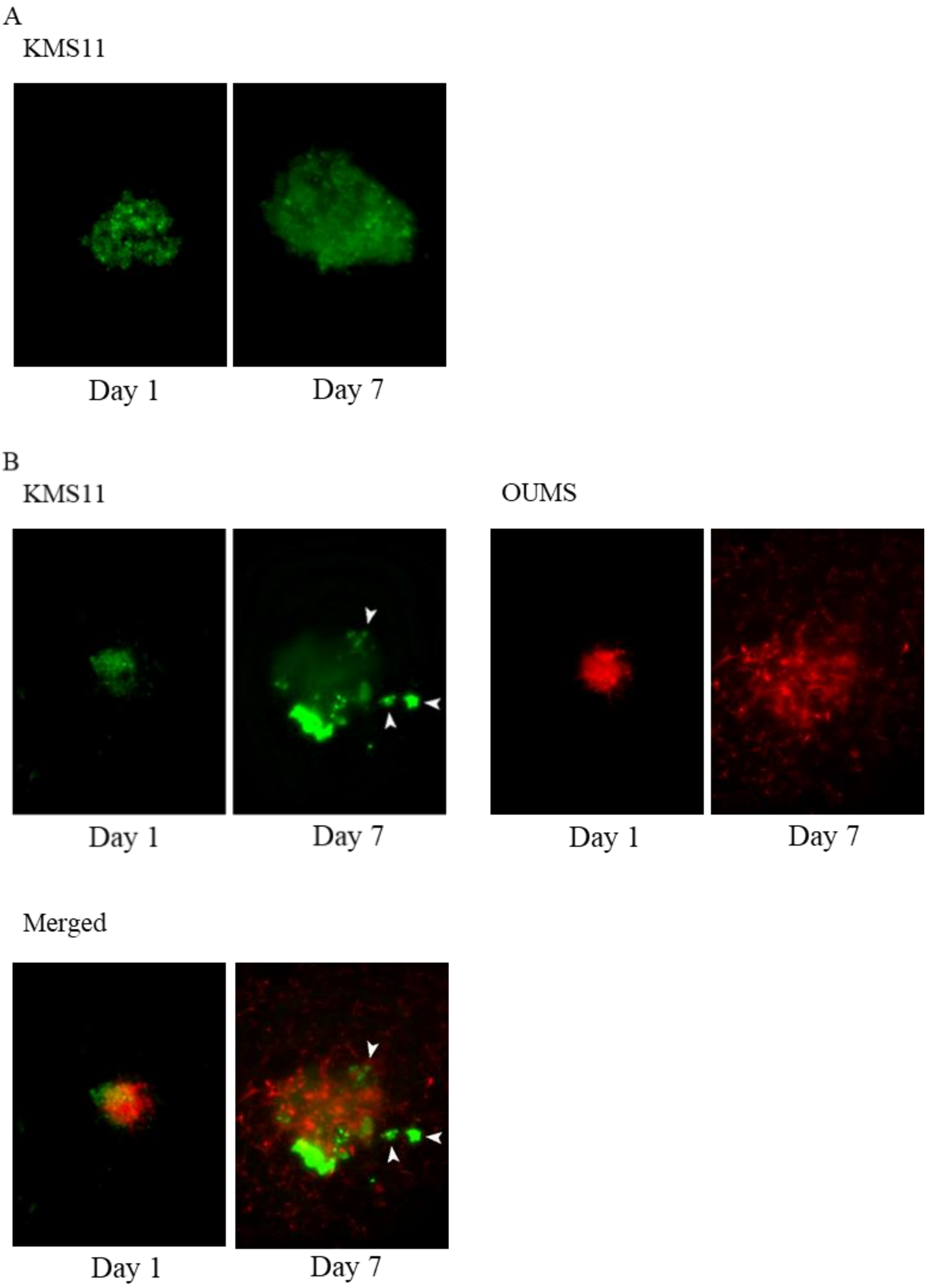

Figure S5. OUMS-36T-3F cells facilitated the invasion of MM cells. (A) Three-dimensional matrix invasion assays of KMS11 MM cells (green color stained with GFP) and (B) KMS11 MM cells (green color stained with GFP) with OUMS-36T-3F fibroblasts (red color stained with m-cherry). White arrows show KMS11 cells (green) migrating after OUMS-36T-3F cells (red).

Figure S6

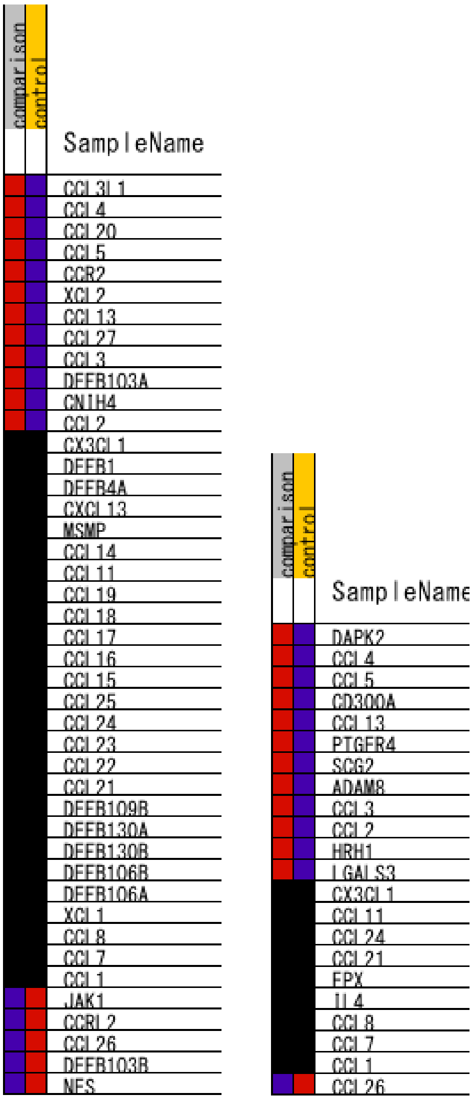

Figure S6 Gene expression in OUMS-36T-3F cells with recombinant TIMP1 (0.5 μg/ml) vs. without recombinant TIMP1. (A) List of genes enriched in the CCR_CHEMOKINE_RECEPTOR_BINDING and the EOSINOPHIL_MIGRATION gene sets derived from Gene Ontology annotations in Gene set enrichment analysis (https://www.gsea-msigdb.org/gsea/msigdb/collection_details.jsp#C5) (Collection 5). The order of genes is ranked according to the running enrichment score.

Video S1 3D matrix invasion assays of KMM1 multiple myeloma cells with OUMS-36T-3F fibroblasts. Invasion of cells into surrounding matrix was photographed every 1 h.
